# Slums, Space, and State of Health—A Link between Settlement Morphology and Health Data

**DOI:** 10.3390/ijerph17062022

**Published:** 2020-03-19

**Authors:** John Friesen, Victoria Friesen, Ingo Dietrich, Peter F. Pelz

**Affiliations:** 1 Chair of Fluid Systems, Technical University of Darmstadt, Otto-Berndt-Str. 2, 64287 Darmstadt, Germany; 2 Klinikum Darmstadt, Grafenstraße 9, 64283 Darmstadt, Germany

**Keywords:** slums, informal settlements, remote sensing, global burden, health data

## Abstract

Approximately 1 billion slum dwellers worldwide are exposed to increased health risks due to their spatial environment. Recent studies have therefore called for the spatial environment to be introduced as a separate dimension in medical studies. Hence, this study investigates how and on which spatial scale relationships between the settlement morphology and the health status of the inhabitants can be identified. To this end, we summarize the current literature on the identification of slums from a geographical perspective and review the current literature on slums and health of the last five years (376 studies) focusing on the considered scales in the studies. We show that the majority of medical studies are restricted to certain geographical regions. It is desirable that the number of studies be adapted to the number of the respective population. On the basis of these studies, we develop a framework to investigate the relationship between space and health. Finally, we apply our methodology to investigate the relationship between the prevalence of slums and different health metrics using data of the global burden of diseases for different prefectures in Brazil on a subnational level.

## 1. Introduction

Worldwide, nearly 1 billion people live in slums and the number is expected to grow further [1]. The living conditions of these people are characterized by, for example, overcrowding, insecure tenure, and/or poor access to infrastructure, such as sanitary facilities. The latter has a considerable impact on the health of the inhabitants [2,3,4]. For this reason, several of the United Nations Sustainable Development Goals mention the inhabitants of slums and the necessary improvement of their living conditions both explicitly (Goal 11—sustainable cities and communities) and implicitly (Goal 3—good health and well being; Goal 6—clean water and sanitation; Goal 9—industry, innovation, and infrastructure). To improve the health situation of the inhabitants of these settlements, studies repeatedly refer to slum upgrading, in which the slum settlements are progressively connected to the infrastructure of the respective city [3,5]. However, before these countermeasures can be initiated, the current situation and developments of these settlements must first be adequately assessed.

In recent years, various studies have repeatedly pointed out that we know relatively little about the urban poor in general [6], and in particular their state of health [2]. To record the current state of a settlement structure is difficult because slums change very quickly. For example, Kit and Lüdeke [7] show that the slum area in Hyderabad, India, increased by 70% from 2003 to 2010.

To map these enormous changes in cities of the Global South as quickly as possible, modern automated methods are needed. Therefore, Lilford et al. [8] point out that the use of earth observation data is a modern way to identify slums and the building environment of their inhabitants, which influences their health conditions. They mention the need for the building environment, which they call “space”, to be explicitly included in the analysis as a risk factor for diseases.

The connection between building environment and health is well known [9]. For example, slum dwellers are more vulnerable to different weather phenomena [10,11] or natural disasters, such as floods [12]. Egondi et al. [13] show that inadequate heating systems during cold periods lead to an increased mortality rate among slum dwellers. Furthermore, the morphology of these settlements leads to an increased danger of heat islands for the inhabitants, which increases with slum size [14]. Furthermore, the shape of the settlements also influences the microclimate, which in turn has an influence on the health of the inhabitants [15].

A recent review by Ige et al. [16] underlines this connection between buildings and health. Unfortunately, slum dwellers with insufficient living conditions (poor access to tap water and sanitary facilities) are only mentioned indirectly in passing. Although a great amount of studies on the impact of the living environment on health in slums is available, the examinations very often take place in very small defined areas (e.g., [17,18,19,20]) and the results are therefore only meaningful on a very small scale and have to be extrapolated to bigger scales.

Nevertheless, we are experiencing a global trend towards the collection of an incredible amount of data with free accessibility, and these data often come from different scientific domains and on various geographic scales. This raises the question of which data in what resolution can be used to answer questions relating public health. Weigand et al. [9] show that socioeconomic and health data are often collected on an individual level or via censuses on a contextual level. Although recent studies highlight the potential of combining geo-information systems (GIS) and health data in general [21], as well as more recently through the use of artificial intelligence [22], this potential has not yet been discussed sufficiently in relation to slums. In this work, we take a step in this direction and show the possibilities related to how spatial data on slums can be linked to health data in order to identify current local health trends and initiate countermeasures.

To this end, we use the following approach: first, we ask how slums in particular and the spatial environment in general have been recorded in recent years and what the advantages and disadvantages of these methods are, focusing on slum detection using earth observation data. Second, we conduct a literature review in PubMed searching for the terms “slums” or “informal settlements”, summarizing the health-related publications on slums of the last five years with a focus on the spatial scale of the studies. On the basis of these two literature reviews, we discuss a framework to investigate the connection between the building environment and the health status of the slum dwellers and discuss how information about the health status of this social group can be collected and what characteristics these data have or could have. In an application example, we investigate the relationship between information on slum dwellers and health metrics in Brazil on a subnational level. Finally, we discuss the results of the study with the abovementioned general analysis to identify possible future trends.

## 2. Reviews on Space and Health

In the following, we first compile current information about slums and their identification. Thereafter, we present the results of a review, examining the latest publications on slums and informal settlements in PubMed. On the basis of this literature review, we discuss how and on which scales it is possible to investigate the relationship between health data and information on settlement morphology.

### 2.1. Information on Space

There are many different names for settlements of the urban poor. The most common terms are “slum” or “informal settlement”, but there are also local specific names in different parts of the world, such as “favela”, “barrio”, “shanty town”, “kampong”, amongst others [23]. Although concrete images and conceptions are often associated with these names, exact quantitative definitions are not yet available [23,24,25]. One definition frequently mentioned in publications is from the UN-Habitat, which describes households belonging to a slum meeting at least one of the following five criteria: non-durable housing, inadequate sanitation, unsafe water, insufficient living space, or insecure tenure [1]. However, this definition has been criticized from a quantitative point of view, as it is not linked to measurable variables [8]. From a qualitative point of view, the nomenclatures “slum”, “informal settlement”, and others have also been criticized, as they often have negative connotations [23]. Therefore, they have been replaced in recent publications by more neutral terms, such as “deprivation areas” [26].

Although the definitions can be criticized and discussed from many angles, the large number of existing studies shows that the urban poor often live in settlements that are morphologically different from their surroundings. The inhabitants of these settlements form a separate social group [27] that is exposed to increased health risks [2], and is associated with insufficient access to infrastructures such as sanitary facilities or water supply and inadequate housing situations. These settlements are often self-built and rarely recognized by the local or national governments [5].

Basically, the aim is therefore to classify these spaces and describe their characteristics. Space can be characterized and classified according to Lilford et al. [8] by surveys or by earth observation images. The advantages and disadvantages of the respective recording types have been discussed in detail in previous publications [23,28,29]. With regard to slums, the use of earth observation data has especially established itself in recent years (cf. [28,29]), since the technical possibilities have expanded enormously. Kohli et al. [30] studied slums and developed an ontology for slums for image classification using remote sensing data.

On the basis of this ontology, Wurm and Taubenböck [27] have shown by comparing earth observation data and census surveys that a special settlement morphology represents a social group. They did an extensive review of the existent literature describing slums as a form of “arrival cities” [31]. While surveys are time-consuming, information obtained by remote sensing can be analyzed very quickly and recent developments can be monitored. Furthermore, a high spatial resolution is possible (down to less than 1 m [28]). The actually existing, physical conditions are mapped and no survey biases arise. However, remote sensing cannot provide any or only insufficient information about the condition of the sanitation supply or the connection to the water supply [23]. Of the five dimensions in the above-mentioned UN definition, only one, non-durable housing, can therefore be covered.

However, recent studies in particular have shown that earth observation data can be used as a basis for recording settlements, which are then enriched with further geo-referenced data, e.g., from social networks or other data sources [32,33], to obtain more comprehensive information about these regions and in particular on their socioeconomic or health status [9].

A problem here is that there is no uniform data collection in which all the available spatial information on slums is recorded, even though efforts to this end have been made [34]. A desirable goal would be a kind of global dataset, similar to the “Global Urban Footprint”, on which the populated area was automatically recorded by Esch et al. [35]. Comparable data can lead to global insights [36], e.g., studies of eight different cities of the Global South show that slums have a similar size [37,38,39]. Before this goal becomes tangible, however, a uniform definition of a slum must be established, which is still under discussion at present [23].

It is clear that the focus should move away from the large known slums to the much more common small slums. Of course, only an automated process can provide this enormous scope. However, there are already approaches that can be used in the future. Aside from the discussion on the definition of slums, the focuses of recent publications are the temporal change of slums [40,41] and the adaption of machine-learning methods [26,40,42,43], which automatically record slums combining remote sensing data with other sources [23]. Remote sensing datasets are also used to improve infrastructure planning for slums [44,45].

**Figure 1:**
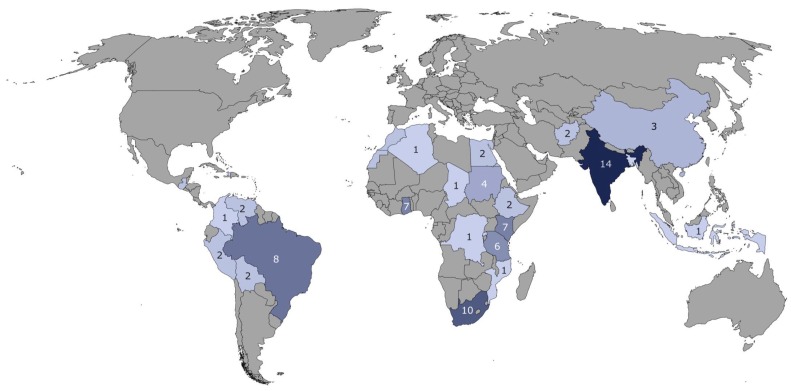
Visualization of the number of studies on slum identification by remote sensing data. We used data from Mahabir et al. [29] to create the map.

Looking at the geographical distribution of the slum classifications carried out in recent years, it is striking that this does not reflect the distribution of the slum population estimated worldwide [29]. The distribution of studies on slum identification is shown in Figure 1, which we formulated from the information in the study by Mahabir et al. [29].

Especially in the case of sub-Saharan Africa, where the largest population increase is expected in the coming years, with a huge amount of this happing in slums [46], further studies are necessary to assess the living situation of the inhabitants. This is particularly difficult because in many cities, it is very difficult to distinguish between formal and informal settlements [28].

### 2.2. Information on Health

The literature on the relationship between *slums* and *health* has produced some reviews in recent years. The connection between slums and health was generally pointed out [2,3,47], as well as the connection between slum upgrading and health [5]. It was also shown that the physical environment and infrastructure intervention, such as road paving or water supply, have significant effects on the health of slum dwellers [48]. Abdi et al. [49] describe what kind of diseases should be investigated in slums (diabetes, hypertension, dengue fever, and anaemia). They refer to slums in Bangalore, India. Our aim is to generalize this approach to a global scale.

As stated above, Lilford et al. [8] postulated in a recent study that space should be considered in health studies. On the basis of this work, we ask what global regions and on which scales studies on health in slums are conducted. Unfortunately, there is no database with information on slum health on a global scale. Therefore, we searched PubMed for journal articles with the keywords *slums* or *informal settlements*. Although, as mentioned above, other terms may also be used, these two terms are those most commonly used in literature and by the UN. We limited the keyword search to title and abstract, and we restricted the search to English articles from the last five years. Even though other platforms like Scopus or WebofScience also have an enormous amount of data, we chose PubMed because it has the largest amount of medical literature [50], it is preferred for the research of biomedical studies [51], and it is free to use [50].

Searching for the terms *slums* or *informal settlements*, we found 415 articles. Of these, 11 were not related to the health of the slum dwellers, 17 others referred to results of SLUMS (Saint Louis University Mental Status), a test used to quantify dementia. Of the remaining 387 publications, 10 were comments and one was an erratum, which did not contain specific information or the text was not accessible.

**Figure 2:**
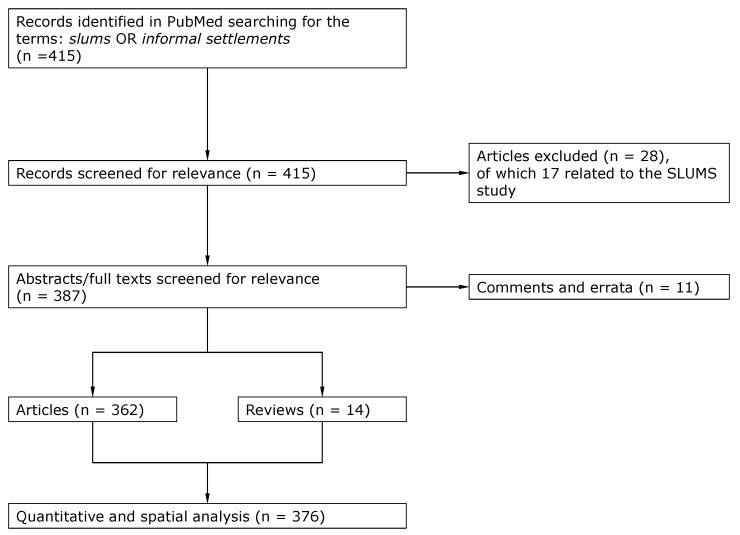
This figure shows the flowchart according to the adapted PRISMA guideline [52].

The remaining publications included 14 reviews, mostly discussing problems on a global scale, and 362 articles. The flowchart according the adapted PRISMA guideline [52] is shown in Figure 2. We classified these 376 publications both spatially and medically using the categories shown in Table 1.

The division into spatial scales was done using the following procedure: If one or two slums were mentioned in the study, we referred to them as *slum* specific studies. If more than two slums were examined in a study or if no specific number of slums were mentioned, we assigned the study to the *city* scale. If more than one city was examined and this or a specific region was explicitly mentioned, we designated the study as *sub-national*. If there was no specific information on city or region, the study was assigned to the *country* class. If more than one country was examined in the study and the countries were explicitly named, we called the study *cross-national*. Studies that described a topic on a global scale, e.g., reviews, were marked as *global*.

We divided the examined diseases into seven categories: communicable, non-communicable, underweight/malnutrition, sexual and maternal health/behavior, injuries, mental health, and non-specific.

The category *communicable* contains diseases that have the potential to infect other persons mainly through bacteria or viruses, for example diarrhea, tuberculosis, and HIV. All diseases that cannot infect another person directly, like diabetes, cardiovascular/neurological problems, and cancer, are part of the category *non-communicable*. *Underweight and malnutrition* was classified as a category that includes food insecurity, wasting, and stunting, especially in children. The category *sexual and maternal health and behavior* consists of all cases that concern sexual intercourse, contraception, pregnancy, childbirth, and breastfeeding. All injuries caused by external violence, like blows, from weapons, and accidents were included in the category *injuries* (physical trauma). The category *mental health* covers diseases that have an influence on physiological well-being, for example depression, suicidal thoughts, and mourning. We classified as *non-specific* all cases that could not be explicitly placed in one of the other six categories, like general studies on sanitation that do not investigate its influence on a specific infectious disease [53] and fundamental reviews like Abdi et al. [49].

We analyze the results according to different aspects in the following three sections. In the main text, we address examples of the found literature. The full classification can be found in the Supplementary Materials (Table S1).

#### 2.2.1. Analysis by Health Categories

**Figure 3:**
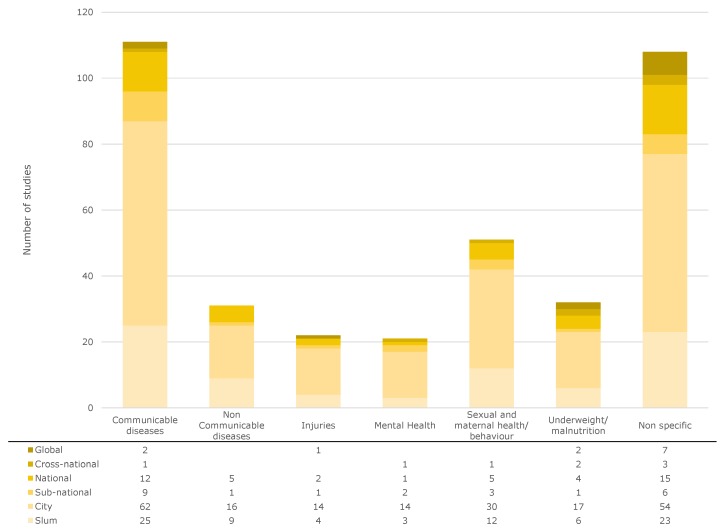
Categorization of the investigated studies into different spatial and medical categories.

In Figure 3, the studies are classified according to the abovementioned categories. All publications analyzed here are listed in the Supplementary Material in an .xlsx file, which lists the spatial scale, the medical category, the geographical entity, and the DOI. This allows the reader to trace all analyses we carried out and to look up the corresponding publications.

As expected, the most frequently investigated class of disease, with 111 studies, is *communicable diseases* (29.5% of all studies). Of these, more than half of the studies (62) were conducted at city level. The communicable diseases most frequently studied in the context of slums were diarrheal diseases (e.g., [54,55]), HIV (e.g., [56,57]), and tuberculosis (e.g., [58,59]), as expected. The communicable diseases are followed by studies on *sexual and maternal behavior* (13.6%). The main focus in these studies was on contraceptive methods [60] or general studies on pregnancy health (e.g., [61,62]). *Nutritional issues* were examined in 8.5% of the studies, mainly with regard to children (e.g., [63,64]). *Non-communicable diseases* (8.2% of all studies) that are examined most frequently are cardiovascular diseases (e.g., [65,66]) or diabetes (e.g., [67,68]). There were only a few studies on cervical cancer (e.g., [69]), confirming a statement in a recent report by the WHO [70] that urges researchers not to neglect cancer in studies on the health of slum dwellers. In addition, there was no study on neurological diseases. With regard to *injury*-related illnesses, which account for 5.9% of all studies, they were almost exclusively conducted on domestic (e.g., [71,72]) or partner-related violence (e.g., [73]). *Mental health* problems represent 5.6% of the total studies. Depression in particular has been studied regularly (e.g., [74]), often in connection with abortion (e.g., [75]). *Non-specific* studies were mentioned 106 times, with most of the studies also being conducted on the city level (50%). These are, for example, studies on aging and the related effects or questions [76,77], studies on the influence of community centers [78], noise exposure [79], the impact of climate change on health [80], the influence of smoke from cooking stations [81], and general studies on sanitation that do not investigate its influence on a specific infectious disease.

Since our investigation focuses on the spatial scales of the studies, we refer to the large studies by Lilford et al. [3] or Corburn and Sverdlik [5], in which the different disease classes are discussed in detail.

It should be noted that even if infectious diseases are by far the greatest risk to the health of slum dwellers today, the study of other diseases should not be neglected. With regard to cancer, the World Health Organization (WHO) pointed out precisely these dangers in its latest report, since a focus on infectious diseases leads to a neglect of non-communicable diseases [70].

We have published the table shown in Figure 3 in the Supplementary Material (Table S1) with references. Table 2 shows an excerpt of the full table.

It can be used to quickly identify PubMed publications of the last five years in regard to health and spatial categories.

#### 2.2.2. Analysis by Region

**Figure 4:**
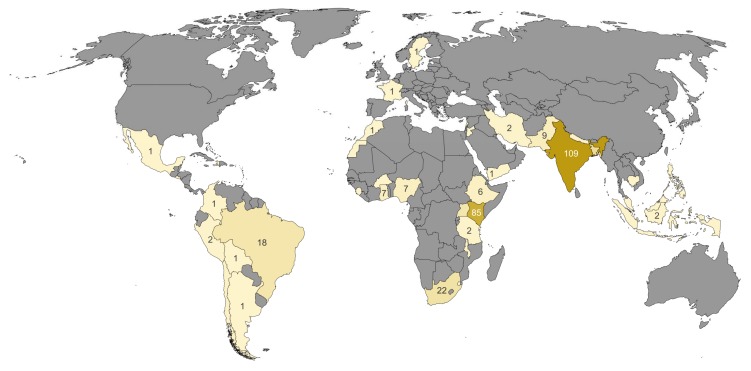
Number of studies on slum health in the respective countries. Cross-national and global studies are not included.

Figure 4 lists the studies on health and slums by country. Cross-national and global studies are not included in this figure, as they account for only a small proportion (5%) of all studies (see Section 2.2.3).

We analyze the studies using the World Bank regions, shown in Table 3. Except for Europe and the Middle East and North Africa, the number of slum dwellers per region is provided by the World Development Indicators (WDI) of the World Bank. Using this information and the 355 studies on the national or lower geographical scale, we calculated the number of studies per 10 million slum dwellers per region.

As expected, almost all studies were conducted in the Global South (Africa, South America, and Asia) [28].

*Europe.* The only two exceptions are the studies on informal settlements of Roma from Sweden and France [102,103].

*Sub-Saharan Africa.* With 7.64 studies per 10 million inhabitants, sub-Saharan Africa has the second highest value. The studies that examine slum health in sub-Saharan Africa (SSA) are mainly conducted in Kenya (85), Nigeria (7), Ghana (7), and Ethiopia (6). The 13 countries where the studies were conducted represent about 61% of the population of SSA. Although this is the largest proportion, the question arises whether the results found here can be applied to other countries in these regions. For example, there were no studies on slums in the Democratic Republic of Congo, although by population, it is the fourth largest African country.

**Figure 5:**
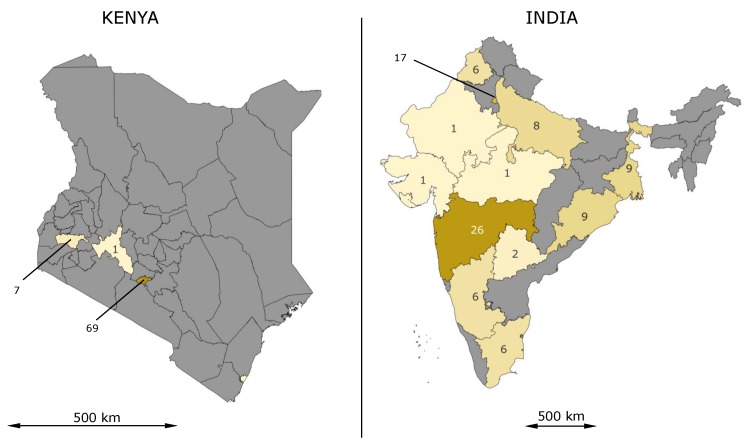
Number of studies on slum health in Kenya (**left**) and India (**right**).

If one takes a closer look at the studies for sub-Saharan Africa on the slum and city level, it is noticeable that by far the most studies were carried out in Kenya (78), especially in Kenya’s capital city Nairobi, and mainly in two specific slums. This fact can be mainly attributed to the fact that in Nairobi, a health study in slums was carried out in which the health conditions in slums were examined [104]. Although Nairobi is by far the largest city in Kenya with 4.4 million inhabitants and accounts for about 10% of the population, the question arises whether the results can be transferred to other parts of the country (cf. Figure 5, left). Even more critical is the question of whether the results of the studies carried out here can be transferred to the whole of sub-Saharan Africa, as the climatic conditions in this huge region differ considerably from one another.

A second example for a sub-Saharan country is Nigeria, which has a population of about 206 million people according to current UN estimates (2020). Six out of the seven mentioned studies were conducted for either Lagos or Enugu. Even with conservative population estimates for these cities, more than 50% of the population is not represented.

Similar to the study of Mahabir et al. [29], it can be seen that the largest share of studies refers to well-known cities with slums, e.g., Nairobi in Kenya. Kenya and especially Nairobi is strongly overrepresented in the studies. The proportion of people in Nairobi, Kenya is marginal compared to all sub-Saharan Africa. The question is whether the living conditions and the illness conditions of the slums in Lagos can be compared with the environmental conditions of the slums in Nairobi.

*South Asia.* In comparison to its population, South Asia has the highest number of studies per slum inhabitant (Table 3). Most of the studies were conducted in India (109) and Bangladesh (30). In contrast to the high number of studies for the slums of Nairobi, the medical studies for India are spread across the whole country (cf. Figure 5, right), although the districts in the west are more strongly represented than those in the east. The two most populated cities Mumbai (21 studies) and Delhi (17 studies) have the largest number of studies in India. In five of the 12 districts where studies were conducted, more than one city was studied. In the neighboring Bangladesh, 30 studies were conducted, 22 of them in the capital, Dhaka. There is a similar concentration of studies in the capital as in Kenya. Further studies were conducted for Nepal (3) and Pakistan (9).

Compared with the African continent, which has a similar population size to India and Bangladesh, the studies are thus more evenly distributed and more in proportion to the population size.

*Latin America and Caribbean*. Looking at the number of studies per 10 million slum inhabitant of 3.2, it is about half as large for the Latin America and Caribbean region as for South Asia and sub-Saharan Africa. There are studies for nine different countries Argentina, Bolivia, Brazil, Chile, Columbia, El Salvador, Haiti, Mexico, and Peru, and most of the studies are conducted in Brazil (18 studies). Both the studies within Brazil and in the entire Latin American region are relatively evenly distributed, as can be seen in Figure 4.

*East Asia and Pacific*. The case of East Asia is very interesting. The World Bank estimates that there are about 325 million slum dwellers in this region. Although the number of slum dwellers in China is estimated to be about 180 million, our search conditions did not identify any studies for this country. Studies were conducted in only six countries Cambodia, Indonesia, Malaysia, Melanesia, Pacific Islands, and Philippines. The number of studies per 10 million slum dwellers has the lowest value for this region (0.22).

*Middle East and North Africa*. Seven studies in five countries (Iran, Jordan, Lebanon, Morocco, and Yemen) have been carried out for this region, one in North Africa and the other six in the Middle East. Several of those studies relate to temporary informal settlements resulting from the armed conflicts in this region [98,105].

#### 2.2.3. Analysis by Scale

**Figure 6:**
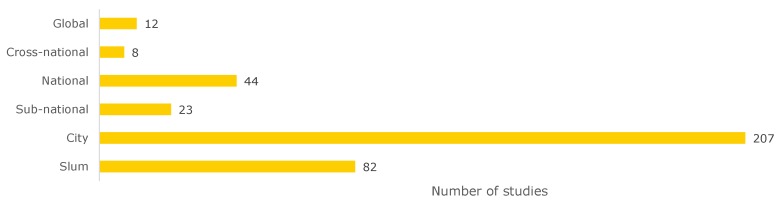
Number of studies on slum health per geographical scale.

**Figure A1:**
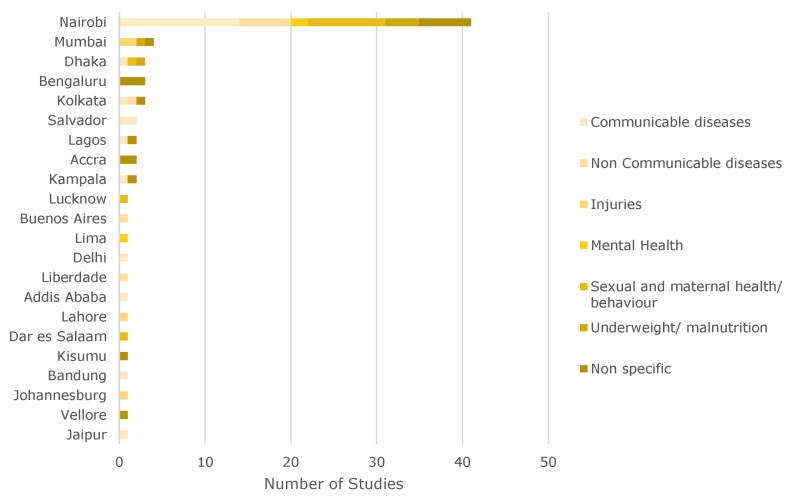
This figure shows the number of studies per city on slum scale. The colors label the health categories of the studies.

Another way to analyze the literature is to look at the scales the slum health studies cover (Figure 6). About 21.9% of the studies examined diseases at the *slum* level. Of the 82 studies conducted at the slum level, 41 were located in Nairobi (50%), which means that most of the information on diseases on the slum level published in the last five years is limited to one city in eastern Africa. A breakdown by city can be seen in Figure A1 in Appendix A.

**Figure A2:**
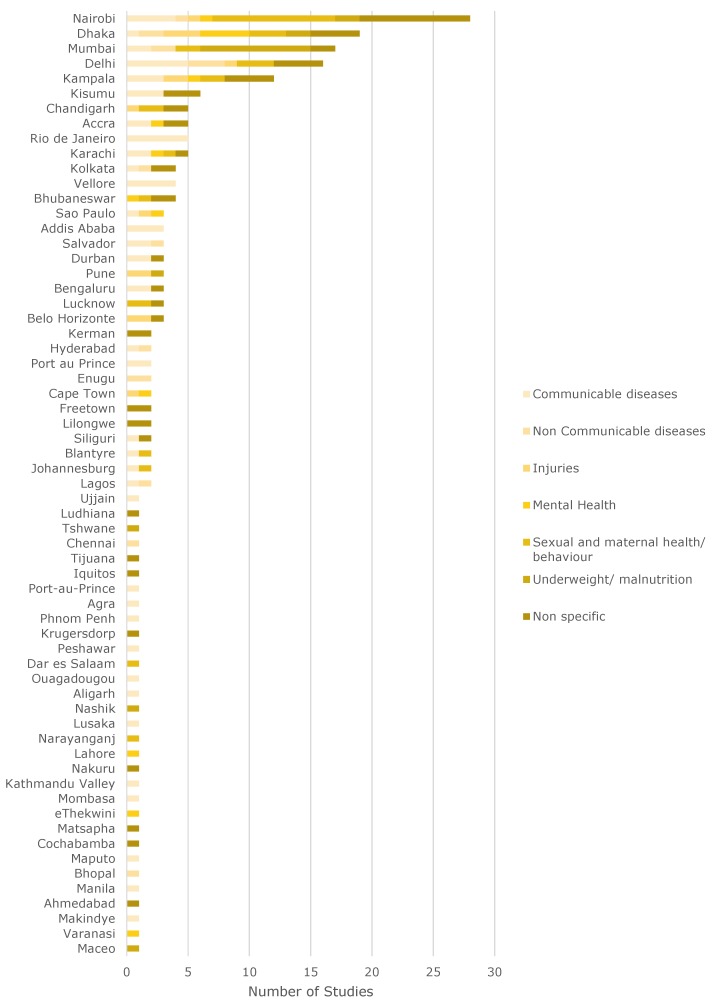
This figure shows the number of studies per city on city scale. The colors label the health categories of the studies.

The geographical scale of cities has been studied the most. About half of all studies (55.2%) were conducted on the *city* level. Of these, about one third (36%) were conducted in sub-Saharan Africa (28 in Nairobi, 12 in Kampala, 6 in Kisumu). All these cities are located in East Africa. Compared to their size, megacities such as Lagos, Nigeria (two studies) or Kinshasa, Democratic Republic of Congo (no study) are severely underrepresented. About 25% (40 studies) were conducted in South Asia (19 Dhaka, 17 Mumbai, 16 Delhi). Here again, the three cities are in India or Bangladesh. The studies with their respective disease classes are listed in Figure A2.

Out of the 23 *sub-national* studies, 14 were conducted in South Asia (10 India, 2 in Nepal, 1 in Bangladesh and in Pakistan), 5 in sub-Saharan Africa (4 in South Africa and 1 in Ethiopia), and 4 in Chile.

The 44 studies on *national* scales have been conducted mainly in India (13), Kenya (7), and South Africa (7).

Looking at the *cross-national* scale, only eight studies are available. There is one cross-national study on communicable diseases [84] in Burkina Faso and Kenya, one on malnutrition between Chile and Kenya [106], one on nurturing care in Angola and Kenya [107], one on contraceptive methods in Bangladesh and Kenya [108], one on early child loss in South Africa and India [109], and three non-specific on noise annoyance and sensitivity [110] between dwellers of informal settlements in South Africa and inhabitants of Switzerland, one on community health in three Melanesian countries [111], and one on urban growth and water access in sub-Saharan Africa [112].

On a *global* level, there are different reviews focusing on different aspects: van de Vijver et al. [113] review health programs in slums, Corburn and Sverdlik [5] slum upgrading, Ezeh et al. [2] look at the history of these settlement forms, as well as social and geographical factors. Oliver et al. [114] focus on water quality, Nelson et al. [115] vaccinations for children, and Goudet et al. [116] examine articles concerning malnutrition of children in slums.

In the context of this analysis, it must also be mentioned that the titles of the studies were often misleading, for example, by referring to countries when in the end only two slums in a city were examined (e.g., [117]).

### 2.3. Conclusion of Reviews

We have seen that a lot of data are available, but only in very specific regions. The geographical scales most frequently studied are the city and slum level. What we also see clearly is that programs in which health data are recorded are very important. The program in Nairobi had a huge impact on our results [104]. If a lot of information is available in a particular region or city, it is useful to analyze it, which leads to a high number of studies, but new studies are also developed to investigate the interaction with the results found there.

Often analyses in geography [118] and medicine [2] focus on the largest urban entities on different scales. Thus, studies are mainly conducted in well-known cities, such as Dhaka, Mumbai, or Nairobi, and in well-known slums, such as Kibera in Nairobi or Dharavi in Mumbai. However, this does not take into account the majority of the population, since urbanization processes mainly take place in medium-sized cities [118] or medium-sized slums [37].

The health status of slums is developing. If the focus of monitoring and observation is only on specific cities and their intervention programs for health improvement (see Nairobi), processes that occur in the immediate neighborhood could be overlooked.

Sub-national and cross-national studies are only available in very small numbers, and intervention measures should take place on all regional scales. Therefore, information should also be available on all scales in order to be able to implement specific measures. What is often missing are studies that show sub-national differences (see application study) as well as studies that carry out cross-national investigations.

It should be investigated whether specific morphological characteristics correlate with specific settlement morphologies. On the basis of the results discussed so far, this is possible in detail, especially for Nairobi and specific regions of India, e.g., Mumbai or Delhi, since both settlement data and a larger number of studies on slum health are available for these cities.

## 3. Conceptual Framework

Various publications have pointed out that slum dwellers are more vulnerable and exposed to increased risks due to their living conditions [2,5]. In order to investigate the relationship between settlement morphology and health data, information is required from both domains. We therefore have two types of data initially:

(i) Data on the settlement morphology or the inhabitants of a specific spatial environment (number of inhabitants in an environment, population density, number of slum dwellers in relation to formal inhabitants in a defined area, spatial form of a slum, distance to the nearest infrastructure, etc.) or physical parameters of the respective slums, such as floor space, building volume, slope inclination, etc;
(ii) Data available on the health status, risks on other health metrics of the inhabitants of a defined area (Figure 4 and Figure 5).

Since we know from the various studies mentioned above that the environment has an influence on people’s health, and this is even more so in the case of slums due to poor connection to the infrastructure and the resulting reduced hygiene, it is necessary to examine the correlations between these two data sources more closely.

The ideal situation would be that uniform information on health data of different health classes as well as data on the physically existing building structure would be available for all relevant places in the world at a high resolution. Previous studies that have investigated the link between poor populations and health data have done so at the national, or in two cases, sub-national level [119]. However, this resolution is much too low to detect local effects, for example, when a specific spatial environment exert an effect on the slum dwellers.

Unfortunately, the data are not available in this homogeneity with a high resolution. We rather see an enormous heterogeneity in the geographical as well as in the health data. The morphological data have been collected by different methods (e.g., census, earth observation [6]), and therefore the results of the studies are not always comparable. At the same time, there are great challenges in the determination of slum data for areas of sub-Saharan Africa, since formal and informal settlements do not differ that much, and the question arises whether a binary distinction makes sense at all [8].

On the other hand, there are studies on different diseases but no general databases with different disease classes, e.g., according to the classification above, on the health of slums with a very high resolution.

On the basis of these two findings, one can conclude that the ideal state, as described above, does not yet exist. An exception based on our reviews would be Nairobi, Kenya, or Mumbai, India, since very detailed studies have been carried out there on different disease classes and at the same time remote sensing studies on slum topography are also available. On the other hand, the health information still needs to be processed.

Although there have been efforts to collect data, e.g., by the Institute of Health Metrics and Evaluation (IHME), with the aim of providing health data up to a resolution of 5 km × 5 km or finer (in the mission statement of the IHME, Seattle, Washington, Director Christopher Murray says that a spatial resolution of 5 km × 5 km or better is targeted), the question arises how to investigate information about the connection between slum development and health data up to this point and on the other hand, up to which scale the data must be resolved.

Here, information from remote sensing is suitable as a data basis, since characteristics can be investigated using globally uniform methods. If one assumes that the physical structure has an influence on the health of the inhabitants, then at least a resolution at settlement level is advantageous. Several studies have shown that slums often have a size of about 0.01 km^2^ [37,38,39]. On the basis of these studies, the spatial resolution of the health metrics in cities should be increased by at least a factor of 100 to reflect the similar size of slums mentioned above, whose health outcomes vary enormously from formal settlements.

However, since this would require an enormous effort, alternative frameworks should be developed, which we cover in the following.

The higher the resolution of the data, the more accurately disease trends can be represented and local phenomena identified. Le Comber et al. [120] introduced a method several years ago to identify hot spots for infectious diseases similar to criminal geographic profiling. The high spatial and temporal resolution achieved by using modern methods (see below) allows continuous monitoring of health and settlement morphological metrics. If, for example, an increase in cases of illness is detected in a specific area, it can be investigated whether these correlate with settlement morphological conditions in the surrounding area.

**Figure 7:**
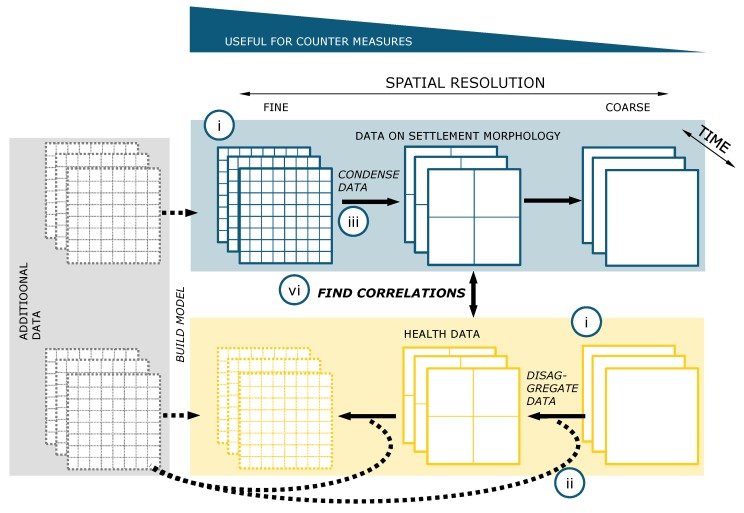
Framework to investigate correlations between settlement (blue box) and health data (yellow box). Furthermore, it is possible to enrich the different settlement and health data with additional data to increase the resolution of the datasets. Examples of additional data are described in the text. Only three spatial resolutions are shown representatively, of course an unlimited number of intermediate levels is possible. The numbering in the blue circles shown in the figure is explained in detail in the text.

The goal of the framework presented in this paper is to investigate the relationship between health data on the one hand, and morphological data on the other hand, on an identical scale. If this is not available, investigations can be carried out using the following framework or workflow, which is shown in Figure 7. The following points refer to the points in the blue circles shown in Figure 7.

(i) Find the best health and settlement morphological data for the region of interest in the best possible data quality and resolution;
(ii) *Try to disaggregate the spatial resolution of your coarser dataset by adding additional information* (grey box in Figure 7). This additional information could be, for example, geo-referenced and anonymized health data from hospitals, like in the study of [121], where information from hospitals is used to find regional dependencies or search queries from mobile phones modulated to health data via models or otherwise [122]. A step forward in this direction was made by Maina et al. [123] by providing geo-referenced data of hospitals in sub-Saharan Africa. Although these models are subject to uncertainties, they can help to disaggregate data to a higher spatial resolution;
(iii) *Bring the other data source to the same resolution.* That means either improvement using models, or if the data are available at a much higher resolution, they should be condensed; for example, by describing two-dimensional data about distribution moments. When data are available only in a fine-resolution, they can be condensed to larger scales in a variety of ways. Spatially high-resolution data form a spatial distribution in a coarse-resolution area and can thus be described by different moments characterizing the corresponding distribution. For example, the number of inhabitants can be related to the detected slum area to determine a population density. Even independently of inhabitants, the settlement morphology alone can be analyzed, for example, by determining the proportion of slum area per urban area. Furthermore, publicly accessible data such as the Open Street Map can be used to determine the distance between the slum and the nearest doctor or pharmacy;
(iv) *Investigate the relationship between the two information strands by using the abovementioned metrics.* Different statistical methods are known for this purpose, which can range from simple investigations of the correlation to complex machine-learning (e.g., support vector machines, random forest, etc. [46]) models.

The metrics presented here are only a selection that can be expanded as required. The aim here is to describe the reality of the life of the inhabitants as quantitatively as possible using appropriate metrics.

## 4. Application Study

In the following, we explain the methods described above using an example study. The section is divided according to the framework presented in Figure 7.

### 4.1. Find the Best Data on Health and Settlement Morphology

On the basis of our framework, we investigate the relationship between information on slum dwellers and health data for the prefectures in Brazil in an application example. Snyder et al. [124] showed on the basis of census data for Rio de Janeiro that the inhabitants of slums are exposed to increased health risks. We would like to extend this question in a modified form to the country Brazil by investigating to what extent metrics describing urban poverty correlate with health metrics.

### 4.2. Try to Disaggregate the Spatial Resolution of the Health Data

For the health data, we rely on the platform of the Institute of Health Metrics and Evaluation (IHME) [125]. The IHME provide health data on a global, national, and regional scale. Detailed information on the methods used in the Global Burden Disease Study to collect the data can be found in the respective publications in the *Lancet*, e.g., [126]. It should be mentioned that the data of the IHME itself are the results of a model. Health data were disaggregated from coarser spatial resolutions to finer scales or presented with models. The data were thus modeled on the basis of the framework (Figure 6).

The data describe causes of death, prevalence, and other health metrics for different countries and regions. For Brazil, these data are available at the sub-national level for the 26 states (prefectures). Data on prevalence are available for 281 diseases and on deaths for 222 diseases on a sub-national level. A list of the diseases can be found in the Supplementary Material. The data are provided with an error interval. However, we limit ourselves to the mean value, as our aim is only to present a conceptual context and not a quantitative analysis. According to the abovementioned categorization, the settlement data are available on the sub-national level.

### 4.3. Bring the Geographical Data to the Same Resolution

There are different studies that have investigated settlements in Brazilian cities (e.g., [27,38]). Unfortunately, this information is mostly limited to Rio de Janeiro and Sao Paulo. In order to be able to investigate the connection between information on settlement morphology and health metrics, data are needed that are available for all prefectures in Brazil at any given time. This is the case at the time of the last census, which recorded the number of slum dwellers and the area of the slums. The information was collected by the Instituto Brasileiro de Geografia e Estaistica (IBGE), the Brazilian statistical authority, and is also provided by it. In the last census in 2010, the IBGE collected information on slums in Brazil, called sub-normal agglomerations or *Aglomerados subnormais*. These settlement units contain at least 51 houses or shacks and fulfill other criteria [127]. The IBGE on the one hand offers information on the population of the different slums and on the other hand geo-referenced data in shapefiles. A link to the data source is provided in Table A3 in Appendix A. According to the abovementioned categorization, the settlement data are available on the slum level.

We use the information provided here to calculate two metrics. On the one hand, the share of slum dwellers in the total population, and on the other hand, the population density of slum dwellers, calculated for the respective regions. With this method, we condense our data to the resolution of the health data.

### 4.4. Investigate the Relationship between Both Data Types

In order to investigate relationships between the different data sources, we use basic methods of data analysis. We calculate the correlation coefficient according to Pearson ρ between a metric of the settlement morphological data (percentage of slum dwellers of the total population, slum dwellers per km^2^) and a metric of the health data (prevalence of different diseases, proportion of all death of different diseases) listed by the IHME.

Since the spatial resolution of the data is not high, we only investigate linear dependencies between diseases and the share of slum dwellers in the total population. We map the diseases using different measures. These are prevalence and the proportion of deaths.

### 4.5. Results

**Figure 8:**
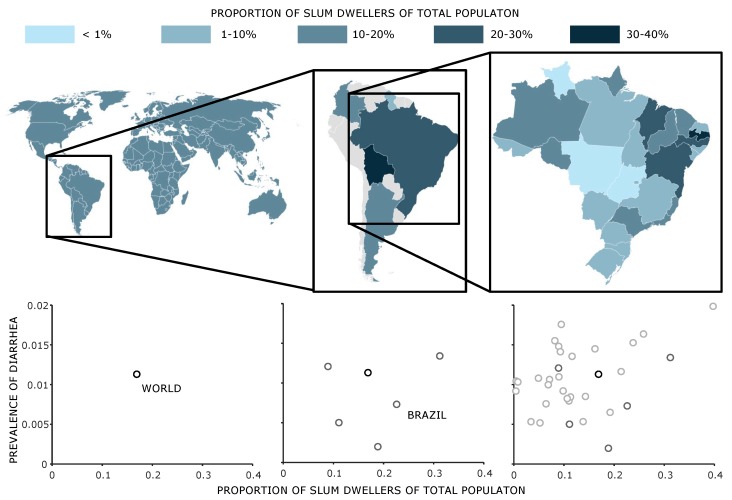
Graphical visualization of the proportion of slum dwellers of the total population for the world (**left**), different South American states (**middle**), and the 26 districts of Brazil (**right**). For the grey shaded countries in South America, no information on the slum population is available. The scatter plots in the lower row show the prevalence of diarrheal diseases in the respective areas (World, South America, Brazil) on the Y-axis above the proportion of slum dwellers of the total population on the X-axis.

First, we put the results into a global context. For this purpose, we used World Bank data to graphically illustrate the share of the slum population of the total population worldwide and for countries in South America alongside the share of the slum population in the total population for the different Brazilian prefectures (Figure 8, upper).

At the global level, the proportion of slum dwellers was 16.9% in 2009 (The latest World Bank data on slum population are from 2014 and estimate the proportion of slum population at 15.9% of the world population. Since we want to compare the global and national data of the countries of South America with the regional data from Brazil for the year 2010, we use the date (2009), which is closest to the census). However, the share of the slum population in South American states varies between 8.9% (Guyana) and 31.2% (Bolivia). In Brazil, 22.6% of the total population lived in slums in 2009. Here too, the scale can be further refined, ranging from 39% in Para to 0.4% in Roraima when looking at the data on a sub-national level. In Brazil, the proportion of slum dwellers in 22 states is thus below the global average.

If these values, which describe the population situation, are now assigned to the proportion of deaths in the respective region or country, local characteristics can be identified. Using data from the IHME, we plotted the prevalence of diarrheal diseases over the proportion of slum dwellers in the lower diagrams in Figure 8. The higher the spatial resolution of the data, the more likely it is that relationships between the morphology of the settlements and the occurrence of diseases can be described. We see, for example, that at the national level, no correlation can be determined between prevalence of diarrheal diseases and share of slum population. At the sub-national level in Brazil, the two values are moderately positively correlated (ρ=0.509, p=0.0067).

Another advantage of this approach is that local characteristics can be better recorded and whether the housing situation has a fundamental influence on the occurrence of a disease or only represents a secondary risk factor can be identified.

**Figure 9:**
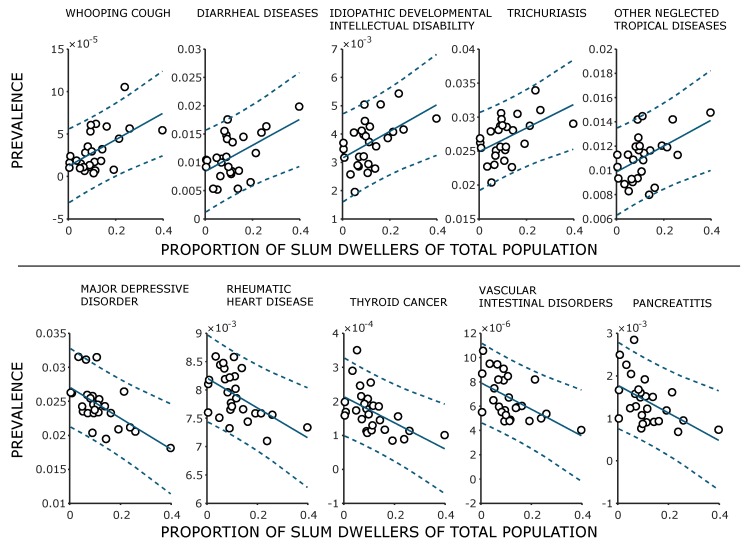
Prevalence of each of the five diseases that show the strongest positive and negative correlation with the proportion of slum population per region. The dots represent the data points of the 27 regions of Brazil, the blue line represents a linear regression through the dots, and the dotted line represents the 95% interval of the regression.

Figure 9 shows the five diseases with the highest and lowest correlation coefficients. The *p*-value is below 1% for all diseases considered here. The graphs also show linear regressions with a 95% prediction interval. Three of the most positively correlated diseases are infectious diseases, one is a disease caused by a worm, and the third is a developmental disorder. These results reflect recent research by Ezeh et al. [2]. In contrast, we see a decreasing prevalence of typical “wealth diseases”, such as depression, cardiovascular diseases, and cancer, with an increasing proportion of slum population. For detailed information on the burden of diseases in Brazil, see Marinho et al. [128]. Detailed information on the correlation coefficients can be found in Table A1 and Table A2 with a short explanation.

The interrelationships very clearly illustrate the fact that Brazil was an emerging economy in 2010, where some regions have high life expectancies while others have rather low life expectancies. The spatial resolution of the data used here is not high. It must of course be taken into account that data used to model disease information can also correlate with morphology data and therefore the respective models must be taken into account.

The results presented here clearly show the advantage of high-resolution data on settlement morphology and health metrics. On the one hand, it is possible to record the relationship between different settlement morphological parameters and health metrics, and on the other hand, it is possible to record local deviations from global trends.

However, it must also be mentioned that we lose information by condensing the data. We compare data from slums in a region with data from formal settlements in the same region. The results should therefore not be interpreted causally, but only show the correlations found with the corresponding methodology.

A list of the health metrics of the diseases (prevalence, proportion of causes of death) that correlated most closely with the metrics studied (proportion of slum dwellers and slum dwellers per km^2^) is provided in the Appendix A. The results presented here should therefore be understood primarily as an illustration of the methods and the framework. The results themselves confirm previously findings, as shown for example by Lilford et al. [3] or Corburn and Sverdlik [5].

## 5. Discussion

It is known from literature that the environment has an enormous influence on human health [9]. This is particularly the case in slums or informal settlements, since the physical environment and the associated lack of access to infrastructure significantly increase the risk of diseases [2,3]. It is therefore of utmost importance to record the current state of health of the slum population and its living conditions.

In other studies, classes of disease and previous knowledge about slums have been collected [2,3,48,49]. However, this study is the first to examine the existing literature in its spatial extent and distribution. Furthermore, the question arises as to how the studies in the respective disease categories (communicable, non-communicable, etc.) are geographically distributed. This investigation would have exceeded the scope of this work, but can be carried out using the list provided in the Supplementary Material. Moreover, how medical care is provided in the slum area should be examined in more detail and on a regionally dependent basis. This would be a further link between geographical and medical data.

Snyder et al. [124] explicitly investigated the different health risks of inhabitants of formal and non-formal settlements in Rio de Janeiro. This type of study is also necessary for other regions of the world with regard to different classes of diseases [8]. In particular, because slum dwellers are exposed to increased risks, also with regard to climatic changes Chersich et al. [80] like more frequent heat waves [15], scarcity of water [112], the threat of flooding [12], it is important to investigate the connections between the physical building structure and health in more detail and to elaborate regional differences.

## 6. Conclusions

In the reviews presented in this paper, it became clear that our knowledge about the life of the 1 billion slum dwellers worldwide is very limited. Both studies on the settlement morphology of slum dwellers and on health aspects focus strongly on specific regions, cities, or slums. Many studies were conducted in Nairobi, Kenya. This raises the question of the extent to which the results found can be extrapolated to other regions. Particularly, studies that investigate sub-national or cross-national differences are necessary, as both global trends and local particularities can be identified. According to our review, these studies are underrepresented in the literature.

Furthermore, databases similar to those of the IHME are necessary both with regard to the uniform recording of settlement metrics and with regard to health metrics, in order to be able to investigate global trends and local effects in the interaction of these types of information.

While the aim with regard to geographical studies must above all be the automation of processes in order to be able to map slums uniformly on a global scale, the focus in medical studies should be primarily on comparative work in order to identify regional differences. Furthermore, types of diseases that have not been considered in medical studies so far, such as neurological diseases, should be taken into account.

Despite there being many ways of recording slums globally using remote-sensing methods and enriching this information with additional data sources, there are no uniform databases available so far. Applying our results to geographical and health data in Brazil, we thus had to rely on census data in our study, which are outdated. Although a further census is planned in the near future, which will allow us to examine the temporal development of the results presented here, it will only provide a retrospective view of the possible developments on large temporal scales. Especially in view of the increased risk of slum dwellers to diseases, monitoring with a higher temporal resolution would be desirable.

Uniform data are required, especially for smaller cities, both from a medical and a geographical point of view. Only in this way can the variations in the relationships between settlement structure and health data (which are only hinted at on a very large scale in our results) be recorded in an appropriate manner and then examined on this basis.

## Figures and Tables

**Table 1:** The literature on health was classified using the following classes.

Classification of Space	Health
Slum	Communicable Diseases
City	Non-communicable Diseases
Sub-national	Injuries
National	Mental health
Cross-national	Sexual and maternal health/behavior
Global	Underweight and malnutrition
	Non-specific

**Table 2:** Example of a classified list of references using the introduced spatial and health categories. The full list can be found in the Supplementary Materials Table S1.

	Communicable	Non-Communicable	…
**Global**	[82,83]	-	…
**Cross-National**	[84]	-	…
**National**	[85,86,87,88,89,90,91,92,93,94,95,96]	[97,98,99,100,101]	…
…	…	…	…

**Table 3:** This table shows the 355 studies on the national or smaller scale and the slum population using data from the World Development Indicators (WDI) (2014).

Region	Population Living in Slums in mio.	Number of Studies	Studies Per 10 Million Slum Dwellers
Europe	-	2	-
Middle East and North Africa	-	7	-
Latin America and Caribbean	100.12	32	3.20
South Asia	170.40	151	8.88
sub-Saharan Africa	204.00	156	7.64
East Asia and Pacific	324.98	7	0.22

## Supplementary Materials

The following are available online at https://www.mdpi.com/1660-4601/17/6/2022/s1, 1. Excel File to browse all reviewed references with medical and geographical categories. 2. Document with Table S1: All references classified by health and spatial categories. 3. Textfile with all references and related .bibtex information.

## Abbreviations

The following abbreviations are used in this manuscript:

GIS	geo-information systems
IBGE	Instituto Brasileiro de Geografia e Estaistica
IHME	Institute of Health Metrics and Evaluation
SSA	sub-Saharan Africa
UN	United Nations
WDI	World Development Indicators
WHO	World Health Organisation

## Appendix A

### Appendix A.2. Detailed Information of the Application Study

The results in Table A1 and Table A2 show that there is a moderate correlation between settlement metrics and health data (ρ≈0.5). The *p*-values are usually smaller than 1%, which means that the correlation is significantly different from 0. Despite this correlation, no causal link between the increased proportion of slum dwellers and the health metrics can be identified, so we do not discuss the individual diseases in more detail. The correlations could also be related to other co-factors in the respective prefectures that are not included in the data. For example, climatic, geographical, or economic conditions could both favor slum growth and contribute to an increased spread of infectious diseases (cf. Table A1 and Table A2). More precise results would be obtained by comparing slum and non-slum inhabitants. This would be the task of corresponding studies, which we explicitly do not carry out here, as we focus on an evaluation of the literature. An example of such a comparative study was conducted in Rio de Janeiro by Snyder et al. [124]. The tables listed here are primarily intended to illustrate how both types of data can be linked.

**Table A1:** This table shows the correlation coefficients and related p-values between prevalence or percentage of all deaths and the proportion of slum dwellers of the total population.

Prevalence					
Positive Correlation			Negative Correlation		
Disease	Corr.	*p*-Value	Disease	Corr.	*p*-Value
Whooping cough	0.554	0.0027	Major depressive disorder	−0.591	0.0012
Diarrheal diseases	0.509	0.0067	Rheumatic heart disease	−0.538	0.0038
Idiopathic developmental intellectual disability	0.492	0.0092	Thyroid cancer	−0.530	0.0044
Trichuriasis	0.491	0.0092	Vascular intestinal disorders	−0.527	0.0048
Other neglected tropical diseases	0.483	0.0108	Pancreatitis	−0.512	0.0064
**Deaths**					
**Positive Correlation**			**Negative Correlation**		
**Disease**	**Corr.**	***p*** **-Value**	**Disease**	**Corr.**	***p*** **-Value**
Hemolytic disease and other neonatal jaundice	0.569	0.0020	Other cardiomyopathy	−0.498	0.0082
Acute glomerulonephritis	0.553	0.0027	Pancreatitis	−0.495	0.0086
Drug-susceptible tuberculosis	0.525	0.0049	Thyroid cancer	−0.491	0.0094
Other intestinal infectious diseases	0.498	0.0081	Alcoholic cardiomyopathy	−0.482	0.0109
Extensively drug-resistant tuberculosis	0.485	0.0104	Vascular intestinal disorders	−0.477	0.0119

**Table A2:** This table shows the correlation coefficients and related p-values between prevalence or percentage of all deaths and the numbers of slum dwellers per km^2^.

Prevalence					
Positive Correlation			Negative Correlation		
Disease	Corr.	*p*-Value	Disease	Corr.	*p*-Value
Asbestosis	0.613	0.0007	Maternal sepsis and other maternal infections	−0.469	0.0135
Coal workers pneumoconiosis	0.535	0.0041	Urinary tract infections	−0.465	0.0145
Glaucoma’	0.492	0.0092	Ectopic pregnancy	−0.449	0.0188
Cataract’	0.478	0.0118	Other gynecological diseases	−0.436	0.0231
Migraine’	0.474	0.0125	Neonatal sepsis and other neonatal infections	−0.411	0.0334
**Deaths**					
**Positive Correlation**			**Negative Correlation**		
**Disease**	**Corr.**	***p*** **-Value**	**Disease**	**Corr.**	***p*** **-Value**
Coal workers pneumoconiosis	0.532	0.0043	Poisoning by other means	−0.546	0.0032
Ischemic stroke	0.442	0.0211	Physical violence by other means	−0.523	0.0051
Alzheimer”s disease and other dementias	0.424	0.0274	Non-venomous animal contact	−0.518	0.0056
Non-melanoma skin cancer (squamous-cell carcinoma)	0.385	0.0471	Chronic kidney disease due to glomerulonephritis	−0.512	0.0064
Silicosis	0.351	0.0727	Chronic kidney disease due to other and unspecified causes	−0.505	0.0072

**Table A3:** Examples for health and settlement data.

Health Data		
Description	Source	Years
Health data for US	[129]	different datasets
WHO	[130]	different datasets
IHME	[131]	1990–2017
World Bank	[132]	1960–2019
**Settlement Data**		
**Description**	**Source**	**Years**
Information on subnormal agglomerates from Brazilian census	[133]	2010
Information on land use in Nairobi, Kenya 2010	[134]	2010
Information on slums in Dhaka, Bangladesh	[135]	2006 & 2010
Information on slums in Hyderabad, India	[7]	2003 & 2010
Information on land use in Ouagadougou, Burkina Faso	[136]	2018
Information on land use in Dakar, Senegal	[136]	2018
