# Supplementary material for: Slums, Space, and State of Health—A Link between Settlement Morphology and Health Data
Source: Int J Environ Res Public Health. 2020 Mar 19;17(6):2022. doi: 10.3390/ijerph17062022 (PMC7143924; doi:10.3390/ijerph17062022)
Supplement: Supplementary file 1 [file ijerph-17-02022-s001.zip › Supplementary Material/Supplementary_Material.pdf]

Supplementary Materials: Slums, Space and State of Health - A Link Between Settlement Morphology and Health Data

John Friesen [1], Victoria Friesen [2], Ingo Dietrich[1], and Peter F. Pelz [1,*]

1. Classified publications

In the following Table S1 the 376 studies we investigated in our medical review are classified according to different spatial and health categories.

Table S1. All references classified by health and spatial categories.

	communi-cable	non communi-cable	injuries	mental health	mal-nutrition	sexual and maternal health	non specific
**Global**	[1,2]	-	[3]	-	[4,5]	-	[6–12]
**Cross-National**	[13]	-	-	[14]	[15,16]	[17]	[18–20]
**Country**	[21–32]	[33–37]	[38,39]	[40]	[41–44]	[45–49]	[50–64]
**Sub-National**	[65–73]	[74]	[75]	[76,77]	[78]	[79–81]	[82–87]
**City**	[88–149]	[150–165]	[166–179]	[180–193]	[194–210]	[211–240]	[241–294]
**Slum**	[295–319]	[320–328]	[329–332]	[333–335]	[336–341]	[342–353]	[354–376]

References

1. Gibbs, A. Tackling gender inequalities and intimate partner violence in the response to HIV: moving towards effective interventions in Southern and Eastern Africa. *African Journal of AIDS Research* **2016**, *15*, 141–148. doi:10.2989/16085906.2016.1204331.
2. Luby, S.P. Urban Slums: A Supportive Ecosystem for TyphoidalSalmonellae. *The Journal of Infectious Diseases* **2018**, *218*, S250–S254. doi:10.1093/infdis/jiy324.
3. Twigg, J.; Christie, N.; Haworth, J.; Osuteye, E.; Skarlatidou, A. Improved Methods for Fire Risk Assessment in Low-Income and Informal Settlements. *International Journal of Environmental Research and Public Health* **2017**, *14*, 139. doi:10.3390/ijerph14020139.
4. Kontopodis, M. How and why should children eat fruit and vegetables? Ethnographic insights into diverse body pedagogies. *Social Science & Medicine* **2015**, *143*, 297–303. doi:10.1016/j.socscimed.2014.10.062.
5. Goudet, S.; Griffiths, P.; Bogin, B.; Madise, N. Interventions to tackle malnutrition and its risk factors in children living in slums: a scoping review. *Annals of Human Biology* **2016**, *44*, 1–10. doi:10.1080/03014460.2016.1205660.
6. Lilford, R.J.; Oyebode, O.; Satterthwaite, D.; Melendez-Torres, G.J.; Chen, Y.F.; Mberu, B.; Watson, S.I.; Sartori, J.; Ndugwa, R.; Caiaffa, W.; Haregu, T.; Capon, A.; Saith, R.; Ezeh, A. Improving the health and welfare of people who live in slums. *The Lancet* **2017**, *389*, 559–570. doi:10.1016/s0140-6736(16)31848-7.
7. Ezeh, A.; Oyebode, O.; Satterthwaite, D.; Chen, Y.F.; Ndugwa, R.; Sartori, J.; Mberu, B.; Melendez-Torres, G.J.; Haregu, T.; Watson, S.I.; Caiaffa, W.; Capon, A.; Lilford, R.J. The history, geography, and sociology of slums and the health problems of people who live in slums. *The Lancet* **2017**, *389*, 547–558. doi:10.1016/s0140-6736(16)31650-6.
8. Nelson, K.N.; Wallace, A.S.; Sodha, S.V.; Daniels, D.; Dietz, V. Assessing strategies for increasing urban routine immunization coverage of childhood vaccines in low and middle-income countries: A systematic review of peer-reviewed literature. *Vaccine* **2016**, *34*, 5495–5503. doi:10.1016/j.vaccine.2016.09.038.
9. Corburn, J.; Sverdlik, A. Slum Upgrading and Health Equity. *International Journal of Environmental Research and Public Health* **2017**, *14*, 342. doi:10.3390/ijerph14040342.
10. Crocker-Buque, T.; Mindra, G.; Duncan, R.; Mounier-Jack, S. Immunization, urbanization and slums - a systematic review of factors and interventions. *BMC Public Health* **2017**, *17*. doi:10.1186/s12889-017-4473-7.

11. McGee, J.A.; Ergas, C.; Greiner, P.T.; Clement, M.T. How do slums change the relationship between urbanization and the carbon intensity of well-being? *PLOS ONE* **2017**, *12*, e0189024. doi:10.1371/journal.pone.0189024.
12. Oliver, S.; Corburn, J.; Ribeiro, H. Challenges Regarding Water Quality of Eutrophic Reservoirs in Urban Landscapes: A Mapping Literature Review. *International Journal of Environmental Research and Public Health* **2018**, *16*, 40. doi:10.3390/ijerph16010040.
13. Soura, A.B.; Mberu, B.; Elungata, P.; Lankoande, B.; Millogo, R.; Beguy, D.; Compaore, Y. Understanding Inequities in Child Vaccination Rates among the Urban Poor: Evidence from Nairobi and Ouagadougou Health and Demographic Surveillance Systems. *Journal of Urban Health* **2014**, *92*, 39–54. doi:10.1007/s11524-014-9908-1.
14. Goldstein, R.D.; Lederman, R.I.; Lichtenthal, W.G.; Morris, S.E.; Human, M.; Elliott, A.J.; Tobacco, D.; Angal, J.; Odendaal, H.; Kinney, H.C.; and, H.G.P. The Grief of Mothers After the Sudden Unexpected Death of Their Infants. *Pediatrics* **2018**, *141*, e20173651. doi:10.1542/peds.2017-3651.
15. Pridmore, P.; Carr-Hill, R.; Amuyunzu-Nyamongo, M.; Lang'o, D.; McCowan, T.; Charnes, G. Tackling the Urban Health Divide Though Enabling Intersectoral Action on Malnutrition in Chile and Kenya. *Journal of Urban Health* **2015**, *92*, 313–321. doi:10.1007/s11524-015-9942-7.
16. Richter, L.M.; Lye, S.J.; Proulx, K. Nurturing Care for Young Children under Conditions of Fragility and Conflict. *New Directions for Child and Adolescent Development* **2018**, *2018*, 13–26. doi:10.1002/cad.20232.
17. Machiyama, K.; Huda, F.A.; Ahmmed, F.; Odwe, G.; Obare, F.; Mumah, J.N.; Wamukoya, M.; Casterline, J.B.; Cleland, J. Women's attitudes and beliefs towards specific contraceptive methods in Bangladesh and Kenya. *Reproductive Health* **2018**, *15*. doi:10.1186/s12978-018-0514-7.
18. Barrington, D.; Sridharan, S.; Saunders, S.; Souter, R.; Bartram, J.; Shields, K.; Meo, S.; Kearton, A.; Hughes, R. Improving community health through marketing exchanges: A participatory action research study on water, sanitation, and hygiene in three Melanesian countries. *Social Science & Medicine* **2016**, *171*, 84–93. doi:10.1016/j.socscimed.2016.11.003.
19. Sieber, C.; Ragettli, M.S.; Brink, M.; Olaniyan, T.; Baatjies, R.; Saucy, A.; Vienneau, D.; Probst-Hensch, N.; Dalvie, M.A.; Röösli, M. Comparison of sensitivity and annoyance to road traffic and community noise between a South African and a Swiss population sample. *Environmental Pollution* **2018**, *241*, 1056–1062. doi:10.1016/j.envpol.2018.06.007.
20. Dos Santos, S.; Adams, E.; Neville, G.; Wada, Y.; de Sherbinin, A.; Mullin Bernhardt, E.; Adamo, S. Urban growth and water access in sub-Saharan Africa: Progress, challenges, and emerging research directions. *Science of The Total Environment* **2017**, *607-608*, 497–508. doi:10.1016/j.scitotenv.2017.06.157.
21. Selmouni, F.; Zidouh, A.; Nejjari, C.; Bekkali, R. Acceptability of the human papilloma virus vaccine among Moroccan parents: a population-based cross-sectional study. *Eastern Mediterranean Health Journal* **2015**, *21*, 555–563. doi:10.26719/2015.21.8.555.
22. Ogbudebe, C.L.; Chukwu, J.N.; Nwafor, C.C.; Meka, A.O.; Ekeke, N.; Madichie, N.O.; Anyim, M.C.; Osakwe, C.; Onyeonoro, U.; Ukwaja, K.N.; Oshi, D.C. Reaching the underserved: Active tuberculosis case finding in urban slums in southeastern Nigeria. *International Journal of Mycobacteriology* **2015**, *4*, 18–24. doi:10.1016/j.ijmyco.2014.12.007.
23. Lau, C.L.; Watson, C.H.; Lowry, J.H.; David, M.C.; Craig, S.B.; Wynwood, S.J.; Kama, M.; Nilles, E.J. Human Leptospirosis Infection in Fiji: An Eco-epidemiological Approach to Identifying Risk Factors and Environmental Drivers for Transmission. *PLOS Neglected Tropical Diseases* **2016**, *10*, e0004405. doi:10.1371/journal.pntd.0004405.
24. Hoy-Schulz, Y.E.; Jannat, K.; Roberts, T.; Zaidi, S.H.; Unicomb, L.; Luby, S.; Parsonnet, J. Safety and acceptability of Lactobacillus reuteri DSM 17938 and Bifidobacterium longum subspecies infantis 35624 in Bangladeshi infants: a phase I randomized clinical trial. *BMC Complementary and Alternative Medicine* **2015**, *16*. doi:10.1186/s12906-016-1016-1.
25. Mbae, C.; Mulinge, E.; Guleid, F.; Wainaina, J.; Waruru, A.; Njiru, Z.K.; Kariuki, S. Molecular Characterization of Giardia duodenalis in Children in Kenya. *BMC Infectious Diseases* **2016**, *16*. doi:10.1186/s12879-016-1436-z.
26. McLaren, Z.M.; Schnippel, K.; Sharp, A. A Data-Driven Evaluation of the Stop TB Global Partnership Strategy of Targeting Key Populations at Greater Risk for Tuberculosis. *PLOS ONE* **2016**, *11*, e0163083. doi:10.1371/journal.pone.0163083.

27. Jennings, L.; Mathai, M.; Linnemayr, S.; Trujillo, A.; Mak'anyengo, M.; Montgomery, B.E.E.; Kerrigan, D.L. Economic Context and HIV Vulnerability in Adolescents and Young Adults Living in Urban Slums in Kenya: A Qualitative Analysis Based on Scarcity Theory. *AIDS and Behavior* **2017**, *21*, 2784–2798. doi:10.1007/s10461-017-1676-y.
28. Sahimin, N.; Lim, Y.A.L.; Douadi, B.; Khalid, M.K.N.M.; Wilson, J.J.; Behnke, J.M.; Zain, S.N.M. Hookworm infections among migrant workers in Malaysia: Molecular identification of Necator americanus and Ancylostoma duodenale. *Acta Tropica* **2017**, *173*, 109–115. doi:10.1016/j.actatropica.2017.06.011.
29. Singh, S.; Sahu, D.; Agrawal, A.; Vashi, M.D. Ensuring childhood vaccination among slums dwellers under the National Immunization Program in India - Challenges and opportunities. *Preventive Medicine* **2018**, *112*, 54–60. doi:10.1016/j.ypmed.2018.04.002.
30. Samuel, S.R.; Muragaboopathy, V.; Patil, S. Transgender HIV status, self-perceived dental care barriers, and residents' stigma, willingness to treat them in a community dental outreach program: Cross-sectional study. *Special Care in Dentistry* **2018**, *38*, 307–312. doi:10.1111/scd.12315.
31. Arias-Orozco, P.; Bástida-González, F.; Cruz, L.; Villatoro, J.; Espinoza, E.; Zárate-Segura, P.B.; Recuenco, S. Spatiotemporal analysis of canine rabies in El Salvador: Violence and poverty as social factors of canine rabies. *PLOS ONE* **2018**, *13*, e0201305. doi:10.1371/journal.pone.0201305.
32. Mutai, W.C.; Muigai, A.W.T.; Waiyaki, P.; Kariuki, S. Multi-drug resistant Salmonella enterica serovar Typhi isolates with reduced susceptibility to ciprofloxacin in Kenya. *BMC Microbiology* **2018**, *18*. doi:10.1186/s12866-018-1332-3.
33. Sethi, S.; Jonsson, R.; Skaff, R.; Tyler, F. Community-Based Noncommunicable Disease Care for Syrian Refugees in Lebanon. *Global Health: Science and Practice* **2017**, *5*, 495–506. doi:10.9745/ghsp-d-17-00043.
34. Normative Data for Thyroid Stimulating Hormone for Screening of Congenital Hypothyroidism. *The Indian Journal of Pediatrics* **2018**, *85*, 941–947. doi:10.1007/s12098-017-2550-7.
35. Lumagbas, L.B.; Coleman, H.L.S.; Bunders, J.; Pariente, A.; Belonje, A.; de Cock Buning, T. Non-communicable diseases in Indian slums: re-framing the Social Determinants of Health. *Global Health Action* **2018**, *11*, 1438840. doi:10.1080/16549716.2018.1438840.
36. Masekameni, M.; Moolla, R.; Gulumian, M.; Brouwer, D. Risk Assessment of Benzene, Toluene, Ethyl Benzene, and Xylene Concentrations from the Combustion of Coal in a Controlled Laboratory Environment. *International Journal of Environmental Research and Public Health* **2018**, *16*, 95. doi:10.3390/ijerph16010095.
37. Hussain, A.; Roy, T.; Ferdausi, N.; Sen, U. Prevalence of childhood ocular morbidity in a peri-urban setting in Bangladesh: a community-based study. *Public Health* **2019**, *170*, 103–112. doi:10.1016/j.puhe.2019.02.026.
38. Muthengi, E.; Gitau, T.; Austrian, K. Is Working Risky or Protective for Married Adolescent Girls in Urban Slums in Kenya? Understanding the Association between Working Status, Savings and Intimate-Partner Violence. *PLOS ONE* **2016**, *11*, e0155988. doi:10.1371/journal.pone.0155988.
39. Alsarhi, K.; Rahma.; Prevoo, M.J.L.; Alink, L.R.A.; Mesman, J. Maternal Harsh Physical Parenting and Behavioral Problems in Children in Religious Families in Yemen. *International Journal of Environmental Research and Public Health* **2019**, *16*, 1485. doi:10.3390/ijerph16091485.
40. Gibbs, A.; Govender, K.; Jewkes, R. An exploratory analysis of factors associated with depression in a vulnerable group of young people living in informal settlements in South Africa. *Global Public Health* **2016**, *13*, 788–803. doi:10.1080/17441692.2016.1214281.
41. Mchiza, Z.; Steyn, N.; Hill, J.; Kruger, A.; Schönfeldt, H.; Nel, J.; Wentzel-Viljoen, E. A Review of Dietary Surveys in the Adult South African Population from 2000 to 2015. *Nutrients* **2015**, *7*, 8227–8250. doi:10.3390/nu7095389.
42. Hoddinott, J.; Karachiwalla, N.I.; Ledlie, N.A.; Roy, S. Adolescent girls infant and young child nutrition knowledge levels and sources differ among rural and urban samples in Bangladesh. *Maternal & Child Nutrition* **2016**, *12*, 885–897. doi:10.1111/mcn.12355.
43. Chanani, S.; Waingankar, A.; More, N.S.; Pantvaidya, S.; Fernandez, A.; Jayaraman, A. Effectiveness of NGO-government partnership to prevent and treat child wasting in urban India. *Maternal & Child Nutrition* **2019**, *15*, e12706. doi:10.1111/mcn.12706.
44. Joshi, A.; Arora, A.; Amadi-Mgbenka, C.; Mittal, N.; Sharma, S.; Malhotra, B.; Grover, A.; Misra, A.; Loomba, M. Burden of household food insecurity in urban slum settings. *PLOS ONE* **2019**, *14*, e0214461. doi:10.1371/journal.pone.0214461.

45. Kamal, S.M.M. Socioeconomic Factors Associated With Contraceptive Use and Method Choice in Urban Slums of Bangladesh. *Asia Pacific Journal of Public Health* **2011**, *27*, NP2661–NP2676. doi:10.1177/1010539511421194.
46. KULATHINAL, S.; SÄÄVÄLÄ, M. FERTILITY INTENTIONS AND EARLY LIFE HEALTH STRESS AMONG WOMEN IN EIGHT INDIAN CITIES: TESTING THE REPRODUCTIVE ACCELERATION HYPOTHESIS. *Journal of Biosocial Science* **2014**, *47*, 632–649. doi:10.1017/s0021932014000261.
47. Warren, C.E.; Abuya, T.; Kanya, L.; Obare, F.; Njuki, R.; Temmerman, M.; Bellows, B. A cross sectional comparison of postnatal care quality in facilities participating in a maternal health voucher program versus non-voucher facilities in Kenya. *BMC Pregnancy and Childbirth* **2015**, *15*. doi:10.1186/s12884-015-0588-y.
48. Marcil, L.; Afsana, K.; Perry, H.B. First Steps in Initiating an Effective Maternal, Neonatal, and Child Health Program in Urban Slums: the BRAC Manoshi Project's Experience with Community Engagement, Social Mapping, and Census Taking in Bangladesh. *Journal of Urban Health* **2016**, *93*, 6–18. doi:10.1007/s11524-016-0026-0.
49. Patel, A.; Kuhite, P.; Puranik, A.; Khan, S.S.; Borkar, J.; Dhande, L. Effectiveness of weekly cell phone counselling calls and daily text messages to improve breastfeeding indicators. *BMC Pediatrics* **2018**, *18*. doi:10.1186/s12887-018-1308-3.
50. Gupta, I.; Mondal, S. Urban health in India: who is responsible? *The International Journal of Health Planning and Management* **2014**, *30*, 192–203. doi:10.1002/hpm.2236.
51. Beyene, A.; Hailu, T.; Faris, K.; Kloos, H. Current state and trends of access to sanitation in Ethiopia and the need to revise indicators to monitor progress in the Post-2015 era. *BMC Public Health* **2015**, *15*. doi:10.1186/s12889-015-1804-4.
52. Buigut, S.; Ettarh, R.; Amendah, D.D. Catastrophic health expenditure and its determinants in Kenya slum communities. *International Journal for Equity in Health* **2015**, *14*. doi:10.1186/s12939-015-0168-9.
53. Gruebner, O.; Lautenbach, S.; Khan, M.M.H.; Kipruto, S.; Epprecht, M.; Galea, S. Place of Residence Moderates the Risk of Infant Death in Kenya: Evidence from the Most Recent Census 2009. *PLOS ONE* **2015**, *10*, e0139545. doi:10.1371/journal.pone.0139545.
54. Mara, D. Shared sanitation: to include or to exclude? *Transactions of The Royal Society of Tropical Medicine and Hygiene* **2016**, *110*, 265–267. doi:10.1093/trstmh/trw029.
55. Habib, R.R.; Mikati, D.; Hojeij, S.; Asmar, K.E.; Chaaya, M.; Zurayk, R. Associations between poor living conditions and multi-morbidity among Syrian migrant agricultural workers in Lebanon. *The European Journal of Public Health* **2016**, *26*, 1039–1044. doi:10.1093/eurpub/ckw096.
56. Matawle, J.L.; Pervez, S.; Shrivastava, A.; Tiwari, S.; Pant, P.; Deb, M.K.; Bisht, D.S.; Pervez, Y.F. PM2.5 pollution from household solid fuel burning practices in central India: 1. Impact on indoor air quality and associated health risks. *Environmental Geochemistry and Health* **2016**, *39*, 1045–1058. doi:10.1007/s10653-016-9871-8.
57. Nuwasiima, A.; Nuwamanya, E.; Navvuga, P.; Babigumira, J.U.; Asiimwe, F.T.; Lubinga, S.J.; Babigumira, J.B. Study protocol: incentives for increased access to comprehensive family planning for urban youth using a benefits card in Uganda. A quasi-experimental study. *Reproductive Health* **2017**, *14*. doi:10.1186/s12978-017-0400-8.
58. Pörtner, C.C.; hsuan Su, Y. Differences in Child Health Across Rural, Urban, and Slum Areas: Evidence From India. *Demography* **2017**, *55*, 223–247. doi:10.1007/s13524-017-0634-7.
59. Manson, D. Securing a Right to Health:"Integration Villages" and Medical Citizenship of Roma People in France. *Health and human rights* **2017**, *19*, 49.
60. Davis, M.F.; Ryan, N. Inconvenient human rights: Water and sanitation in Sweden's informal Roma settlements. *Health and human rights* **2017**, *19*, 61.
61. Lawana, N.; Booysen, F. Decomposing socioeconomic inequalities in alcohol use by men living in South African urban informal settlements. *BMC Public Health* **2018**, *18*. doi:10.1186/s12889-018-5925-4.
62. Chersich, M.; Wright, C.; Venter, F.; Rees, H.; Scorgie, F.; Erasmus, B. Impacts of Climate Change on Health and Wellbeing in South Africa. *International Journal of Environmental Research and Public Health* **2018**, *15*, 1884. doi:10.3390/ijerph15091884.
63. Chetty, S.; Pillay, L. Assessing the influence of human activities on river health: a case for two South African rivers with differing pollutant sources. *Environmental Monitoring and Assessment* **2019**, *191*. doi:10.1007/s10661-019-7308-4.

64. Magnusson, L.; Ghosh, R.; Jensen, K.R.; Göbel, K.; Wågberg, J.; Wallén, S.; Svensson, A.; Stavenheim, R.; Ahlström, G. Quality of life of prosthetic and orthotic users in South India: a cross-sectional study. *Health and Quality of Life Outcomes* **2019**, *17*. doi:10.1186/s12955-019-1116-y.
65. Lelu, M.; Muñoz-Zanzi, C.; Higgins, B.; Galloway, R. Seroepidemiology of leptospirosis in dogs from rural and slum communities of Los Rios Region, Chile. *BMC Veterinary Research* **2015**, *11*, 31. doi:10.1186/s12917-015-0341-9.
66. Muñoz-Zanzi, C.; Saavedra, F.; Otth, C.; Domancich, L.; Hott, M.; Padula, P. Serological Evidence of Hantavirus Infection in Apparently Healthy People from Rural and Slum Communities in Southern Chile. *Viruses* **2015**, *7*, 2006–2013. doi:10.3390/v7042006.
67. Mason, M.R.; Gonzalez, M.; Hodges, J.S.; Muñoz-Zanzi, C. Protective practices against zoonotic infections among rural and slum communities from South Central Chile. *BMC Public Health* **2015**, *15*. doi:10.1186/s12889-015-1964-2.
68. Mason, M.R.; Encina, C.; Sreevatsan, S.; Muñoz-Zanzi, C. Distribution and Diversity of Pathogenic Leptospira Species in Peri-domestic Surface Waters from South Central Chile. *PLOS Neglected Tropical Diseases* **2016**, *10*, e0004895. doi:10.1371/journal.pntd.0004895.
69. Marimuthu, P. Tuberculosis prevalence and socio-economic differentials in the slums of four metropolitan cities of India. *Indian Journal of Tuberculosis* **2016**, *63*, 167–170. doi:10.1016/j.ijtb.2016.08.007.
70. Fatima, R.; Qadeer, E.; Yaqoob, A.; ul Haq, M.; Majumdar, S.S.; Shewade, H.D.; Stevens, R.; Creswell, J.; Mahmood, N.; Kumar, A.M.V. Extending 'Contact Tracing' into the Community within a 50-Metre Radius of an Index Tuberculosis Patient Using Xpert MTB/RIF in Urban, Pakistan: Did It Increase Case Detection? *PLOS ONE* **2016**, *11*, e0165813. doi:10.1371/journal.pone.0165813.
71. Majorin, F.; Torondel, B.; Routray, P.; Rout, M.; Clasen, T. Identifying Potential Sources of Exposure Along the Child Feces Management Pathway: A Cross-Sectional Study Among Urban Slums in Odisha, India. *The American Journal of Tropical Medicine and Hygiene* **2017**, *97*, 861–869. doi:10.4269/ajtmh.16-0688.
72. Dutta, A.; Pattanaik, S.; Choudhury, R.; Nanda, P.; Sahu, S.; Panigrahi, R.; Padhi, B.K.; Sahoo, K.C.; Mishra, P.R.; Panigrahi, P.; Lekharu, D.; Stevens, R.H. Impact of involvement of non-formal health providers on TB case notification among migrant slum-dwelling populations in Odisha, India. *PLOS ONE* **2018**, *13*, e0196067. doi:10.1371/journal.pone.0196067.
73. Feleke, H.; Medhin, G.; Abebe, A.; Beyene, B.; Kloos, H.; Asrat, D. Enteric pathogens and associated risk factors among under-five children with and without diarrhea in Wegera District, Northwestern Ethiopia. *Pan African Medical Journal* **2018**, *29*, 1–10.
74. Olaniyan, T.; Jeebhay, M.; Röösli, M.; Naidoo, R.; Baatjies, R.; Künzil, N.; Tsai, M.; Davey, M.; de Hoogh, K.; Berman, D.; Parker, B.; Leaner, J.; Dalvie, M.A. A prospective cohort study on ambient air pollution and respiratory morbidities including childhood asthma in adolescents from the western Cape Province: study protocol. *BMC Public Health* **2017**, *17*. doi:10.1186/s12889-017-4726-5.
75. Deuba, K.; Mainali, A.; Alvesson, H.M.; Karki, D.K. Experience of intimate partner violence among young pregnant women in urban slums of Kathmandu Valley, Nepal: a qualitative study. *BMC Women s Health* **2016**, *16*. doi:10.1186/s12905-016-0293-7.
76. Sahoo, K.C.; Hulland, K.R.; Caruso, B.A.; Swain, R.; Freeman, M.C.; Panigrahi, P.; Dreibelbis, R. Sanitation-related psychosocial stress: A grounded theory study of women across the life-course in Odisha, India. *Social Science & Medicine* **2015**, *139*, 80–89. doi:10.1016/j.socscimed.2015.06.031.
77. Hulland, K.R.S.; Chase, R.P.; Caruso, B.A.; Swain, R.; Biswal, B.; Sahoo, K.C.; Panigrahi, P.; Dreibelbis, R. Sanitation, Stress, and Life Stage: A Systematic Data Collection Study among Women in Odisha, India. *PLOS ONE* **2015**, *10*, e0141883. doi:10.1371/journal.pone.0141883.
78. Sibanyoni, J.J.; Tabit, F.T. Assessing the Food Safety Attitudes and Awareness of Managers of School Feeding Programmes in Mpumalanga, South Africa. *Journal of Community Health* **2016**, *42*, 664–673. doi:10.1007/s10900-016-0303-6.
79. Sudhinaraset, M.; Beyeler, N.; Barge, S.; Diamond-Smith, N. Decision-making for delivery location and quality of care among slum-dwellers: a qualitative study in Uttar Pradesh, India. *BMC Pregnancy and Childbirth* **2016**, *16*. doi:10.1186/s12884-016-0942-8.
80. Messinger, C.J.; Mahmud, I.; Kanan, S.; Jahangir, Y.T.; Sarker, M.; Rashid, S.F. Utilization of mobile phones for accessing menstrual regulation services among low-income women in Bangladesh: a qualitative analysis. *Reproductive Health* **2017**, *14*. doi:10.1186/s12978-016-0274-1.

81. Khupakonke, S.; Beke, A.; Amoko, D.H.A. Maternal characteristics and birth outcomes resulting from births before arrival at health facilities in Nkangala District, South Africa: a case control study. *BMC Pregnancy and Childbirth* **2017**, *17*. doi:10.1186/s12884-017-1580-5.
82. Heijnen, M.; Torondel, B.; Clasen, T.; Routray, P. Shared Sanitation Versus Individual Household Latrines in Urban Slums: A Cross-Sectional Study in Orissa, India. *The American Journal of Tropical Medicine and Hygiene* **2015**, *93*, 263–268. doi:10.4269/ajtmh.14-0812.
83. Heijnen, M.; Routray, P.; Torondel, B.; Clasen, T. Neighbour-shared versus communal latrines in urban slums: a cross-sectional study in Orissa, India exploring household demographics, accessibility, privacy, use and cleanliness. *Transactions of The Royal Society of Tropical Medicine and Hygiene* **2015**, *109*, 690–699. doi:10.1093/trstmh/trv082.
84. Shrestha, S.; Shrestha, M.; Wagle, R.R.; Bhandari, G. Predictors of incompletion of immunization among children residing in the slums of Kathmandu valley, Nepal: a case-control study. *BMC Public Health* **2016**, *16*. doi:10.1186/s12889-016-3651-3.
85. Chawla, R.M.; Mitra, P.; Shetiya, S.H.; Agarwal, D.R.; Narayana, D.S.; Bomble, N. Knowledge, Attitude, and Practice of Women in Slums of Pimpri, Chinchwad, Pune, Maharashtra, India, regarding Usage of Mishri. *The Journal of Contemporary Dental Practice* **2017**, *18*, 218–221. doi:10.5005/jp-journals-10024-2020.
86. Sieber, C.; Ragettli, M.S.; Brink, M.; Toyib, O.; Baatjies, R.; Saucy, A.; Probst-Hensch, N.; Dalvie, M.A.; Röösli, M. Land Use Regression Modeling of Outdoor Noise Exposure in Informal Settlements in Western Cape, South Africa. *International Journal of Environmental Research and Public Health* **2017**, *14*, 1262. doi:10.3390/ijerph14101262.
87. Das, M.; Angeli, F.; Krumeich, A.J.S.M.; van Schayck, O.C.P. Patterns of illness disclosure among Indian slum dwellers: a qualitative study. *BMC International Health and Human Rights* **2018**, *18*. doi:10.1186/s12914-018-0142-x.
88. Thomas, R.J.; Ramanujam, K.; Velusamy, V.; Kaliappan, S.P.; Kattula, D.; Muliyil, J.; Kang, G. Comparison of fieldworker interview and a pictorial diary method for recording morbidity of infants in semi-urban slums. *BMC Public Health* **2015**, *15*. doi:10.1186/s12889-015-1372-7.
89. Swahn, M.H.; Culbreth, R.; Salazar, L.F.; Tumwesigye, N.M.; Kasirye, R. Psychosocial correlates of self-reported HIV among youth in the slums of Kampala. *BMC Public Health* **2019**, *19*. doi:10.1186/s12889-019-7480-z.
90. KATTULA, D.; FRANCIS, M.R.; KULINKINA, A.; SARKAR, R.; MOHAN, V.R.; BABJI, S.; WARD, H.D.; KANG, G.; BALRAJ, V.; NAUMOVA, E.N. Environmental predictors of diarrhoeal infection for rural and urban communities in south India in children and adults. *Epidemiology and Infection* **2015**, *143*, 3036–3047. doi:10.1017/s0950268814003562.
91. Hussain, R.S.; McGarvey, S.T.; Fruzzetti, L.M. Partition and Poliomyelitis: An Investigation of the Polio Disparity Affecting Muslims during India s Eradication Program. *PLOS ONE* **2015**, *10*, e0115628. doi:10.1371/journal.pone.0115628.
92. Marlow, M.A.; Maciel, E.L.N.; Sales, C.M.M.; Gomes, T.; Snyder, R.E.; Daumas, R.P.; Riley, L.W. Tuberculosis DALY-Gap: Spatial and Quantitative Comparison of Disease Burden Across Urban Slum and Non-slum Census Tracts. *Journal of Urban Health* **2015**, *92*, 622–634. doi:10.1007/s11524-015-9957-0.
93. Mahapatra, T.; Mahapatra, S.; Banerjee, B.; Mahapatra, U.; Samanta, S.; Pal, D.; Chakraborty, N.D.; Manna, B.; Sur, D.; Kanungo, S. Predictors of Rational Management of Diarrhea in an Endemic Setting: Observation from India. *PLOS ONE* **2015**, *10*, e0123479. doi:10.1371/journal.pone.0123479.
94. Santos, S.D.; de Charles Ouédraogo, F.; Soura, A.B. Water-related factors and childhood diarrhoea in African informal settlements. A cross-sectional study in Ouagadougou (Burkina Faso). *Journal of Water and Health* **2015**, *13*, 562–574. doi:10.2166/wh.2014.115.
95. Balogun, M.R.; Sekoni, A.O.; Meloni, S.T.; Odukoya, O.O.; Onajole, A.T.; Longe-Peters, O.A.; Ogunsola, F.T.; Kanki, P.J. Predictors of tuberculosis knowledge, attitudes and practices in urban slums in Nigeria: a cross-sectional study. *Pan African Medical Journal* **2019**, *32*. doi:10.11604/pamj.2019.32.60.14622.
96. Hemavarneshwari, S.; Shaikh, R.B.; Naik, P.R.; Nagaraja, S.B. Strategy to sensitize private practitioners on RNTCP through medico-social workers in urban field practice area of a Medical College in Bengaluru, Karnataka. *Indian Journal of Tuberculosis* **2019**, *66*, 253–258. doi:10.1016/j.ijtb.2019.04.008.
97. Brown, J.; Cumming, O.; Bartram, J.; Cairncross, S.; Ensink, J.; Holcomb, D.; Knee, J.; Kolsky, P.; Liang, K.; Liang, S.; Nala, R.; Norman, G.; Rheingans, R.; Stewart, J.; Zavale, O.; Zuin, V.; Schmidt, W.P. A controlled,

before-and-after trial of an urban sanitation intervention to reduce enteric infections in children: research protocol for the Maputo Sanitation (MapSan) study, Mozambique. *BMJ Open* **2015**, *5*, e008215–e008215. doi:10.1136/bmjopen-2015-008215.

98. Mathur, A.; Baghel, D.; Jaat, J.; Diwan, V.; Pathak, A. Community-Based Participatory Research and Drug Utilization Research to Improve Childhood Diarrhea Case Management in Ujjain, India: A Cross-Sectional Survey. *International Journal of Environmental Research and Public Health* **2019**, *16*, 1646. doi:10.3390/ijerph16091646.
99. Dako-Gyeke, M.; Kofie, H.M. Factors influencing prevention and control of malaria among pregnant women resident in urban slums, Southern Ghana. *African journal of reproductive health* **2015**, *19*, 44–53.
100. Koenig, S.P.; Rouzier, V.; Vilbrun, S.C.; Morose, W.; Collins, S.E.; Joseph, P.; Decome, D.; Ocheretina, O.; Galbaud, S.; Hashiguchi, L.; Pierrot, J.; Pape, J.W. Tuberculosis in the aftermath of the 2010 earthquake in Haiti. *Bulletin of the World Health Organization* **2015**, *93*, 498–502. doi:10.2471/blt.14.145649.
101. Raoot, A.; Dewan, D.K.; Dubey, A.P.; Batra, R.K.; Seth, S. Measles Outbreak in High Risk Areas of Delhi: Epidemiological Investigation and Laboratory Confirmation. *The Indian Journal of Pediatrics* **2015**, *83*, 200–208. doi:10.1007/s12098-015-1845-9.
102. Sohaila, A.; Shafiq, Y.; Azim, S.; Baloch, B.; Akhtar, A.S.M.; Tikmani, S.S.; Brown, N. Predictors and outcome of tetanus in newborns in slum areas of Karachi City: a case control study. *BMC Research Notes* **2015**, *8*. doi:10.1186/s13104-015-1301-y.
103. Pereira, A.G.L.; de Andrade Medronho, R.; Escosteguy, C.C.; Valencia, L.I.O.; de Avelar Figueiredo Mafra Magalhães, M. Spatial distribution and socioeconomic context of tuberculosis in Rio de Janeiro, Brazil. *Revista de Saúde Pública* **2015**, *49*, 1–8. doi:10.1590/s0034-8910.2015049005470.
104. Wobudeya, E.; Lukoye, D.; Lubega, I.R.; Mugabe, F.; Sekadde, M.; Musoke, P. Epidemiology of tuberculosis in children in Kampala district, Uganda, 2009-2010; a retrospective cross-sectional study. *BMC Public Health* **2015**, *15*. doi:10.1186/s12889-015-2312-2.
105. Mychaleckyj, J.C.; Haque, R.; Carmolli, M.; Zhang, D.; Colgate, E.R.; Nayak, U.; Taniuchi, M.; Dickson, D.; Weldon, W.C.; Oberste, M.S.; Zaman, K.; Houpt, E.R.; Alam, M.; Kirkpatrick, B.D.; Petri, W.A. Effect of substituting IPV for tOPV on immunity to poliovirus in Bangladeshi infants: An open-label randomized controlled trial. *Vaccine* **2016**, *34*, 358–366. doi:10.1016/j.vaccine.2015.11.046.
106. Reddy, S.; Prashanth, R.; Devi, B.Y.; Chugh, N.; Kaur, A.; Thomas, N. Prevalence of oral mucosal lesions among chewing tobacco users: A cross-sectional study. *Indian Journal of Dental Research* **2015**, *26*, 537. doi:10.4103/0970-9290.172083.
107. de O. Menezes, A.P.; Azevedo, J.; Leite, M.C.; Campos, L.C.; Cunha, M.; da Gloria S. Carvalho, M.; Reis, M.G.; Ko, A.I.; Weinberger, D.M.; Ribeiro, G.; Reis, J.N. Nasopharyngeal carriage of Streptococcus pneumoniae among children in an urban setting in Brazil prior to PCV10 introduction. *Vaccine* **2016**, *34*, 791–797. doi:10.1016/j.vaccine.2015.12.042.
108. Morin, V.M.; Ahmad, M.M.; Warnitchai, P. Vulnerability to typhoon hazards in the coastal informal settlements of Metro Manila, the Philippines. *Disasters* **2016**, *40*, 693–719. doi:10.1111/disa.12174.
109. Mathur, M.R.; Tsakos, G.; Parmar, P.; Millett, C.J.; Watt, R.G. Socioeconomic inequalities and determinants of oral hygiene status among Urban Indian adolescents. *Community Dentistry and Oral Epidemiology* **2016**, pp. n/a–n/a. doi:10.1111/cdoe.12212.
110. Hagan, J.E.; Moraga, P.; Costa, F.; Capian, N.; Ribeiro, G.S.; Wunder, E.A.; Felzemburgh, R.D.M.; Reis, R.B.; Nery, N.; Santana, F.S.; Fraga, D.; dos Santos, B.L.; Santos, A.C.; Queiroz, A.; Tassinari, W.; Carvalho, M.S.; Reis, M.G.; Diggle, P.J.; Ko, A.I. Spatiotemporal Determinants of Urban Leptospirosis Transmission: Four-Year Prospective Cohort Study of Slum Residents in Brazil. *PLOS Neglected Tropical Diseases* **2016**, *10*, e0004275. doi:10.1371/journal.pntd.0004275.
111. Mistry, N.; Rangan, S.; Dholakia, Y.; Lobo, E.; Shah, S.; Patil, A. Durations and Delays in Care Seeking, Diagnosis and Treatment Initiation in Uncomplicated Pulmonary Tuberculosis Patients in Mumbai, India. *PLOS ONE* **2016**, *11*, e0152287. doi:10.1371/journal.pone.0152287.
112. van der Kop, M.L.; Thabane, L.; Awiti, P.O.; Muhula, S.; Kyomuhangi, L.B.; Lester, R.T.; Ekström, A.M. Advanced HIV disease at presentation to care in Nairobi, Kenya: late diagnosis or delayed linkage to care?—a cross-sectional study. *BMC Infectious Diseases* **2016**, *16*. doi:10.1186/s12879-016-1500-8.

113. Sarkar, R.; Gladstone, B.P.; Warier, J.P.; Sharma, S.L.; Raman, U.; Muliyil, J.; Kang, G. Rotavirus and other diarrheal disease in a birth cohort from Southern Indian community. *Indian Pediatrics* **2016**, *53*, 583–588. doi:10.1007/s13312-016-0892-2.
114. Odhiambo, G.O.; Musuva, R.M.; Odiere, M.R.; Mwinzi, P.N. Experiences and perspectives of community health workers from implementing treatment for schistosomiasis using the community directed intervention strategy in an informal settlement in Kisumu City, western Kenya. *BMC Public Health* **2016**, *16*. doi:10.1186/s12889-016-3662-0.
115. Kerubo, G.; Amukoye, E.; Niemann, S.; Kariuki, S. Drug susceptibility profiles of pulmonary Mycobacterium tuberculosis isolates from patients in informal urban settlements in Nairobi, Kenya. *BMC Infectious Diseases* **2016**, *16*. doi:10.1186/s12879-016-1920-5.
116. Iravatham, C.C.; Neela, V.S.K.; Valluri, V.L. Identifying and mapping TB hot spots in an urban slum by integratingGeographic positioning system and the local postman - A pilot study. *Indian Journal of Tuberculosis* **2019**, *66*, 203–208. doi:10.1016/j.ijtb.2019.02.008.
117. AHMAD, M.; BAHL, S.; KUNWAR, A. Cross-sectional Serologic Assessment of Immunity to Poliovirus in Differential Risk Areas of India: India Seroprevalence Survey-2014. *Indian pediatrics* **2016**, *53*.
118. de Klerk, J.; Moyer, E. "A Body Like a Baby": Social Self-Care among Older People with Chronic HIV in Mombasa. *Medical Anthropology* **2016**, *36*, 305–318. doi:10.1080/01459740.2016.1235573.
119. Prasad, B.M.; Satyanarayana, S.; Chadha, S.S. Lessons learnt from active tuberculosis case finding in an urban slum setting of Agra city, India. *Indian Journal of Tuberculosis* **2016**, *63*, 199–202. doi:10.1016/j.ijtb.2016.08.006.
120. Curtis, A.; Squires, R.; Rouzier, V.; Pape, J.; Ajayakumar, J.; Bempah, S.; Alam, M.T.; Alam, M.; Rashid, M.; Ali, A.; John Morris, J. Micro-Space Complexity and Context in the Space-Time Variation in Enteric Disease Risk for Three Informal Settlements of Port au Prince, Haiti. *International Journal of Environmental Research and Public Health* **2019**, *16*, 807. doi:10.3390/ijerph16050807.
121. Kattula, D.; Jeyavelu, N.; Prabhakaran, A.D.; Premkumar, P.S.; Velusamy, V.; Venugopal, S.; Geetha, J.C.; Lazarus, R.P.; Das, P.; Nithyanandhan, K.; Gunasekaran, C.; Muliyil, J.; Sarkar, R.; Wanke, C.; Ajjampur, S.S.R.; Babji, S.; Naumova, E.N.; Ward, H.D.; Kang, G. Natural History of Cryptosporidiosis in a Birth Cohort in Southern India. *Clinical Infectious Diseases* **2016**, *64*, 347–354. doi:10.1093/cid/ciw730.
122. Daudé, É.; Mazumdar, S.; Solanki, V. Widespread fear of dengue transmission but poor practices of dengue prevention: A study in the slums of Delhi, India. *PLOS ONE* **2017**, *12*, e0171543. doi:10.1371/journal.pone.0171543.
123. Adane, M.; Mengistie, B.; Mulat, W.; Kloos, H.; Medhin, G. Utilization of health facilities and predictors of health-seeking behavior for under-five children with acute diarrhea in slums of Addis Ababa, Ethiopia: a community-based cross-sectional study. *Journal of Health, Population and Nutrition* **2017**, *36*. doi:10.1186/s41043-017-0085-1.
124. Gibbs, A.; Washington, L.; Willan, S.; Ntini, N.; Khumalo, T.; Mbatha, N.; Sikweyiya, Y.; Shai, N.; Chirwa, E.; Strauss, M.; Ferrari, G.; Jewkes, R. The Stepping Stones and Creating Futures intervention to prevent intimate partner violence and HIV-risk behaviours in Durban, South Africa: study protocol for a cluster randomized control trial, and baseline characteristics. *BMC Public Health* **2017**, *17*. doi:10.1186/s12889-017-4223-x.
125. Scorgie, F.; Vearey, J.; Oliff, M.; Stadler, J.; Venables, E.; Chersich, M.F.; Delany-Moretlwe, S. 'Leaving no one behind': reflections on the design of community-based HIV prevention for migrants in Johannesburg's inner-city hostels and informal settlements. *BMC Public Health* **2017**, *17*. doi:10.1186/s12889-017-4351-3.
126. Adane, M.; Mengistie, B.; Medhin, G.; Kloos, H.; Mulat, W. Piped water supply interruptions and acute diarrhea among under-five children in Addis Ababa slums, Ethiopia: A matched case-control study. *PLOS ONE* **2017**, *12*, e0181516. doi:10.1371/journal.pone.0181516.
127. Carvalho, F.R.; Medeiros, T.; de Oliveira Vianna, R.A.; Douglass-Jaimes, G.; Nunes, P.C.G.; Quintans, M.D.S.; Souza, C.F.; Cavalcanti, S.M.B.; dos Santos, F.B.; de Oliveira, S.A.; Cardoso, C.A.A.; Silva, A.A. Simultaneous circulation of arboviruses and other congenital infections in pregnant women in Rio de Janeiro, Brazil. *Acta Tropica* **2019**, *192*, 49–54. doi:10.1016/j.actatropica.2019.01.020.
128. Ignacio, C.F.; da Silva, M.E.C.; Handam, N.B.; de Fatima Leal Alencar, M.; Sotero-Martins, A.; de Lima Barata, M.M.; de Moraes Neto, A.H.A. Socioenvironmental conditions and intestinal parasitic

infections in Brazilian urban slums: a cross-sectional study. *Revista do Instituto de Medicina Tropical de São Paulo* **2017**, *59*. doi:10.1590/s1678-9946201759056.

129. James, R.; Khim, K.; Boudarene, L.; Yoong, J.; Phalla, C.; Saint, S.; Koeut, P.; Mao, T.E.; Coker, R.; Khan, M.S. Tuberculosis active case finding in Cambodia: a pragmatic, cost-effectiveness comparison of three implementation models. *BMC Infectious Diseases* **2017**, *17*. doi:10.1186/s12879-017-2670-8.
130. Manandhar, K.; Bajcharya, K.; Prajapati, R.; Shrestha, N. Prevalence and Predictors of Incomplete Immunization among Children Residing in the Slums of Kathmandu Valley: A Community Based Door-to-Door Survey. *Kathmandu University medical journal (KUMJ)* **2018**, *16*, 8–13.
131. Siddiqui, M.; Khan, A.A.; Varan, A.K.; Esteves-Jaramillo, A.; Sultana, S.; Ali, A.S.; Zaidi, A.K.; Omer, S.B. Intention to accept pertussis vaccine among pregnant women in Karachi, Pakistan. *Vaccine* **2017**, *35*, 5352–5359. doi:10.1016/j.vaccine.2017.08.033.
132. Rivera, V.R.; Jean-Juste, M.A.; Gluck, S.C.; Reeder, H.T.; Sainristil, J.; Julma, P.; Peck, M.; Joseph, P.; Ocheretina, O.; Perodin, C.; others. Diagnostic yield of active case finding for tuberculosis and HIV at the household level in slums in Haiti. *The International Journal of Tuberculosis and Lung Disease* **2017**, *21*, 1140–1146.
133. Ignacio, C.F.; de Lima Barata, M.M.; de Moraes Neto, A.H.A. The Brazilian Family Health Strategy and the management of intestinal parasitic infections. *Primary Health Care Research & Development* **2017**, *19*, 333–343. doi:10.1017/s146342361700072x.
134. Adane, M.; Mengistie, B.; Mulat, W.; Medhin, G.; Kloos, H. The Most Important Recommended Times of Hand Washing with Soap and Water in Preventing the Occurrence of Acute Diarrhea Among Children Under Five Years of Age in Slums of Addis Ababa, Ethiopia. *Journal of Community Health* **2017**, *43*, 400–405. doi:10.1007/s10900-017-0437-1.
135. Barnes, A.N.; Mumma, J.; Cumming, O. Role, ownership and presence of domestic animals in peri-urban households of Kisumu, Kenya. *Zoonoses and Public Health* **2017**, *65*, 202–214. doi:10.1111/zph.12429.
136. Bhattacherjee, S.; Dasgupta, P.; Mukherjee, A.; Dasgupta, S. Vaccine hesitancy for childhood vaccinations in slum areas of Siliguri, India. *Indian Journal of Public Health* **2018**, *62*, 253. doi:10.4103/ijph.ijph_397_17.
137. Adiga, A.; Chu, S.; Eubank, S.; Kuhlman, C.J.; Lewis, B.; Marathe, A.; Marathe, M.; Nordberg, E.K.; Swarup, S.; Vullikanti, A.; Wilson, M.L. Disparities in spread and control of influenza in slums of Delhi: findings from an agent-based modelling study. *BMJ Open* **2018**, *8*, e017353. doi:10.1136/bmjopen-2017-017353.
138. van der Kop, M.L.; Nagide, P.I.; Thabane, L.; Gelmon, L.; Kyomuhangi, L.B.; Abunah, B.; Ekström, A.M.; Lester, R.T. Retention in clinic versus retention in care during the first year of HIV care in Nairobi, Kenya: a prospective cohort study. *Journal of the International AIDS Society* **2018**, *21*, e25196. doi:10.1002/jia2.25196.
139. van der Kop, M.L.; Muhula, S.; Nagide, P.I.; Thabane, L.; Gelmon, L.; Awiti, P.O.; Abunah, B.; Kyomuhangi, L.B.; Budd, M.A.; Marra, C.; Patel, A.; Karanja, S.; Ojakaa, D.I.; Mills, E.J.; Ekström, A.M.; Lester, R.T. Effect of an interactive text-messaging service on patient retention during the first year of HIV care in Kenya (WelTel Retain): an open-label, randomised parallel-group study. *The Lancet Public Health* **2018**, *3*, e143–e152. doi:10.1016/s2468-2667(17)30239-6.
140. Forson, A.O.; Arthur, I.; Ayeh-Kumi, P.F. The role of family size, employment and education of parents in the prevalence of intestinal parasitic infections in school children in Accra. *PLOS ONE* **2018**, *13*, e0192303. doi:10.1371/journal.pone.0192303.
141. Ssekamatte, T.; Isunju, J.B.; Balugaba, B.E.; Nakirya, D.; Osuret, J.; Mguni, P.; Mugambe, R.; van Vliet, B. Opportunities and barriers to effective operation and maintenance of public toilets in informal settlements: perspectives from toilet operators in Kampala. *International Journal of Environmental Health Research* **2018**, *29*, 359–370. doi:10.1080/09603123.2018.1544610.
142. Fatima, S.H.; Zaidi, F.; Adnan, M.; Ali, A.; Jamal, Q.; Khisroon, M. Rat-bites of an epidemic proportion in Peshawar vale; a GIS based approach in risk assessment. *Environmental Monitoring and Assessment* **2018**, *190*. doi:10.1007/s10661-018-6605-7.
143. Sarin, R.; Vohra, V.; Khalid, U.; Sharma, P.P.; Chadha, V.; Sharada, M. Prevalence of pulmonary tuberculosis among adults in selected slums of Delhi city. *Indian Journal of Tuberculosis* **2018**, *65*, 130–134. doi:10.1016/j.ijtb.2017.08.007.
144. Gibbs, A.; Dunkle, K.; Washington, L.; Willan, S.; Shai, N.; Jewkes, R. Childhood traumas as a risk factor for HIV-risk behaviours amongst young women and men living in urban informal settlements in South Africa: A cross-sectional study. *PLOS ONE* **2018**, *13*, e0195369. doi:10.1371/journal.pone.0195369.

145. Ayllón, T.; Câmara, D.C.P.; Morone, F.C.; da Silva Gonçalves, L.; de Barros, F.S.M.; Brasil, P.; Carvalho, M.S.; Honório, N.A. Dispersion and oviposition of Aedes albopictus in a Brazilian slum: Initial evidence of Asian tiger mosquito domiciliation in urban environments. *PLOS ONE* **2018**, *13*, e0195014. doi:10.1371/journal.pone.0195014.
146. Atim, F.; Nagaddya, T.; Nakaggwa, F.; N-Mboowa, M.G.; Kirabira, P.; Okiria, J.C. Agony resulting from cultural practices of canine bud extraction among children under five years in selected slums of Makindye: a cross sectional study. *BMC Oral Health* **2018**, *18*. doi:10.1186/s12903-018-0599-y.
147. Pugliese-Garcia, M.; Heyerdahl, L.W.; Mwamba, C.; Nkwemu, S.; Chilengi, R.; Demolis, R.; Guillermet, E.; Sharma, A. Factors influencing vaccine acceptance and hesitancy in three informal settlements in Lusaka, Zambia. *Vaccine* **2018**, *36*, 5617–5624. doi:10.1016/j.vaccine.2018.07.042.
148. Monk, E.; Kumwenda, M.; Nliwasa, M.; Mpunga, J.; Corbett, E. Factors affecting tuberculosis health message recall 2 years after active case finding in Blantyre, Malawi. *The International Journal of Tuberculosis and Lung Disease* **2018**, *22*, 1007–1015.
149. Baker, K.K.; Senesac, R.; Sewell, D.; Gupta, A.S.; Cumming, O.; Mumma, J. Fecal Fingerprints of Enteric Pathogen Contamination in Public Environments of Kisumu, Kenya, Associated with Human Sanitation Conditions and Domestic Animals. *Environmental Science & Technology* **2018**, *52*, 10263–10274. doi:10.1021/acs.est.8b01528.
150. van de Vijver, S.; Oti, S.; van Charante, E.; Allender, S.; Foster, C.; Lange, J.; Oldenburg, B.; Kyobutungi, C.; Agyemang, C. Cardiovascular prevention model from Kenyan slums to migrants in the Netherlands. *Globalization and Health* **2015**, *11*, 11. doi:10.1186/s12992-015-0095-y.
151. Unger, A.; .; Felzemburgh, R.D.M.; Snyder, R.E.; Ribeiro, G.S.; Mohr, S.; Costa, V.B.A.; Melendez, A.X.T.O.; Reis, R.B.; Santana, F.S.; Riley, L.W.; Reis, M.G.; Ko, A.I. Hypertension in a Brazilian Urban Slum Population. *Journal of Urban Health* **2015**, *92*, 446–459. doi:10.1007/s11524-015-9956-1.
152. Ezeala-Adikaibe, B.A.; Orjioke, C.; Ekenze, O.S.; Ijoma, U.; Onodugo, O.; Okudo, G.; Okwara, C.; Chime, P.; Mbadiwe, N.; Eddy, A.; Onyekonwu, C.; Onyebueke, G.; Ulasi, I.; Mba, A.U. Population-based prevalence of high blood pressure among adults in an urban slum in Enugu, South East Nigeria. *Journal of Human Hypertension* **2015**, *30*, 285–291. doi:10.1038/jhh.2015.49.
153. Mudgapalli, V.; Sharan, S.; Amadi, C.; Joshi, A. Perception of receiving SMS based health messages among hypertensive individuals in urban slums. *Technology and Health Care* **2016**, *24*, 57–65. doi:10.3233/THC-151097.
154. Ezeala-Adikaibe, B.A.; Orjioke, C.; Ekenze, O.; Ijoma, U.; Onodugo, O.; Molokwu, O.; Chime, P.; Mbadiwe, N.; Aneke, E.; Onyekonwu, C.; Okudo, G.; Okwara, C.; Onyebueke, G.; Ulasi, I. Prevalence of active convulsive epilepsy in an urban slum in Enugu South East Nigeria. *Seizure* **2016**, *35*, 100–105. doi:10.1016/j.seizure.2015.12.010.
155. Banerjee, S.; Mukherjee, T.K.; Basu, S. Prevalence, awareness, and control of hypertension in the slums of Kolkata. *Indian Heart Journal* **2016**, *68*, 286–294. doi:10.1016/j.ihj.2015.09.029.
156. Olubodun, T.; Odukoya, O.O.; Balogun, M.R. Knowledge, attitude and practice of cervical cancer prevention, among women residing in an urban slum in Lagos, South West, Nigeria. *Pan African Medical Journal* **2019**, *32*. doi:10.11604/pamj.2019.32.130.14432.
157. Pregel, A.; Vaughan Gough, T.; Jolley, E.; Buttan, S.; Bhambal, A. Ensuring universal access to eye health in urban slums in the Global South: The case of Bhopal (India). *Stud. Health Technol. Inform* **2016**, *229*, 302–313.
158. Vigneswari, A.; Manikandan, R.; Satyavani, K.; Archana, S.; Rajeswari, R.; Viswanathan, V. Prevalence of risk factors of diabetes among urban poor south Indian population. *J Assoc Physicians India* **2015**, *63*, 32–4.
159. Sunita, M.; Singh, A.K.; Rogye, A.; Sonawane, M.; Gaonkar, R.; Srinivasan, R.; Natarajan, S.; Stevens, F.C.J.; Scherpbier, A.J.J.A.; Kumaramanickavel, G.; McCarty, C. Prevalence of Diabetic Retinopathy in Urban Slums: The Aditya Jyot Diabetic Retinopathy in Urban Mumbai Slums Study—Report 2. *Ophthalmic Epidemiology* **2017**, *24*, 303–310. doi:10.1080/09286586.2017.1290258.
160. Rawal, L.B.; Biswas, T.; Khandker, N.N.; Saha, S.R.; Chowdhury, M.M.B.; Khan, A.N.S.; Chowdhury, E.H.; Renzaho, A. Non-communicable disease (NCD) risk factors and diabetes among adults living in slum areas of Dhaka, Bangladesh. *PLOS ONE* **2017**, *12*, e0184967. doi:10.1371/journal.pone.0184967.
161. Vashist, P.; Misra, V.; Singh, S.; Malhotra, S.; Gupta, V.; Dwivedi, S.; Gupta, S. Awareness and eye health-seeking practices for cataract among urban slum population of Delhi: The North India eye disease awareness study. *Indian Journal of Ophthalmology* **2017**, *65*, 1483. doi:10.4103/ijo.ijo_585_16.

162. Wadhwani, M.; Vashist, P.; Singh, S.S.; Gupta, N.; Malhotra, S.; Gupta, A.; Shukla, P.; Bhardwaj, A.; Gupta, V. Diabetic retinopathy screening programme utilising non-mydriatic fundus imaging in slum populations of New Delhi, India. *Tropical Medicine & International Health* **2018**, *23*, 405–414. doi:10.1111/tmi.13039.
163. Chopra, H.V.; Kehoe, S.H.; Sahariah, S.A.; Sane, H.N.; Cox, V.A.; Tarwade, D.V.; Margetts, B.M.; Potdar, R.D.; Fall, C.H.; Joshi, S.R. Effect of a daily snack containing green leafy vegetables on women's fatty acid status: a randomized controlled trial in Mumbai, India. *Asia Pacific journal of clinical nutrition* **2018**, *27*, 804–817.
164. Poli, U.R.; Gowrishankar, S.; Swain, M.; Jeronimo, J. Triage of Women Testing Positive With the careHPV Test on Self-Collected Vaginal Samples for Cervical Cancer Screening in a Low-Resource Setting. *Journal of Global Oncology* **2018**, pp. 1–7. doi:10.1200/jgo.2016.008078.
165. Sutradhar, I.; Gayen, P.; Hasan, M.; Gupta, R.D.; Roy, T.; Sarker, M. Eye diseases: the neglected health condition among urban slum population of Dhaka, Bangladesh. *BMC Ophthalmology* **2019**, *19*. doi:10.1186/s12886-019-1043-z.
166. Tripathy, N.K.; Jagnoor, J.; Patro, B.K.; Dhillon, M.S.; Kumar, R. Epidemiology of falls among older adults: A cross sectional study from Chandigarh, India. *Injury* **2015**, *46*, 1801–1805. doi:10.1016/j.injury.2015.04.037.
167. de Paula Silva, C.J.; Moura, A.C.M.; Paiva, P.C.P.; Ferreira, R.C.; Silvestrini, R.A.; Vargas, A.M.D.; de Paula, L.P.P.; Naves, M.D.; e Ferreira, E.F. Maxillofacial Injuries as Markers of Interpersonal Violence in Belo Horizonte-Brazil: Analysis of the Socio-Spatial Vulnerability of the Location of Victim's Residences. *PLOS ONE* **2015**, *10*, e0134577. doi:10.1371/journal.pone.0134577.
168. Mathur, M.R.; Watt, R.G.; Millett, C.J.; Parmar, P.; Tsakos, G. Determinants of Socioeconomic Inequalities in Traumatic Dental Injuries among Urban Indian Adolescents. *PLOS ONE* **2015**, *10*, e0140860. doi:10.1371/journal.pone.0140860.
169. Parvin, K.; Sultana, N.; Naved, R.T. Disclosure and help seeking behavior of women exposed to physical spousal violence in Dhaka slums. *BMC Public Health* **2016**, *16*. doi:10.1186/s12889-016-3060-7.
170. do Nascimento, C.F.; Duarte, Y.A.O.; Lebrão, M.L.; Filho, A.D.P.C. Individual and contextual characteristics of indoor and outdoor falls in older residents of São Paulo, Brazil. *Archives of Gerontology and Geriatrics* **2017**, *68*, 119–125. doi:10.1016/j.archger.2016.10.004.
171. Sarnquist, C.; Kang, J.L.; Amuyunzu-Nyamongo, M.; Oguda, G.; Otieno, D.; Mboya, B.; Omondi, N.; Kipkirui, D.; Baiocchi, M. A protocol for a cluster-randomized controlled trial testing an empowerment intervention to prevent sexual assault in upper primary school adolescents in the informal settlements of Nairobi, Kenya. *BMC Public Health* **2019**, *19*. doi:10.1186/s12889-019-7154-x.
172. Wesson, H.K.; Bachani, A.M.; Mtambeka, P.; Schulman, D.; Mavengere, C.; Millar, A.J.W.; Hyder, A.A.; van As, A.B. Changing state of pediatric injuries in South Africa: An analysis of surveillance data from a Pediatric Emergency Department from 2007 to 2011. *Surgery* **2017**, *162*, S4–S11. doi:10.1016/j.surg.2017.03.019.
173. Swahn, M.; Culbreth, R.; Staton, C.; Self-Brown, S.; Kasirye, R. Alcohol-Related Physical Abuse of Children in the Slums of Kampala, Uganda. *International Journal of Environmental Research and Public Health* **2017**, *14*, 1124. doi:10.3390/ijerph14101124.
174. Naved, R.; Rahman, T.; Willan, S.; Jewkes, R.; Gibbs, A. Female garment workers' experiences of violence in their homes and workplaces in Bangladesh: A qualitative study. *Social Science & Medicine* **2018**, *196*, 150–157. doi:10.1016/j.socscimed.2017.11.040.
175. Kalokhe, A.S.; Iyer, S.R.; Kolhe, A.R.; Dhayarkar, S.; Paranjape, A.; del Rio, C.; Stephenson, R.; Sahay, S. Correlates of domestic violence experience among recently-married women residing in slums in Pune, India. *PLOS ONE* **2018**, *13*, e0195152. doi:10.1371/journal.pone.0195152.
176. Kalokhe, A.S.; Iyer, S.R.; Gadhe, K.; Katendra, T.; Paranjape, A.; del Rio, C.; Stephenson, R.; Sahay, S. Correlates of domestic violence perpetration reporting among recently-married men residing in slums in Pune, India. *PLOS ONE* **2018**, *13*, e0197303. doi:10.1371/journal.pone.0197303.
177. Swahn, M.; Culbreth, R.; Tumwesigye, N.; Topalli, V.; Wright, E.; Kasirye, R. Problem Drinking, Alcohol-Related Violence, and Homelessness among Youth Living in the Slums of Kampala, Uganda. *International Journal of Environmental Research and Public Health* **2018**, *15*, 1061. doi:10.3390/ijerph15061061.
178. Naved, R.T.; Mamun, M.A.; Mourin, S.A.; Parvin, K. A cluster randomized controlled trial to assess the impact of SAFE on spousal violence against women and girls in slums of Dhaka, Bangladesh. *PLOS ONE* **2018**, *13*, e0198926. doi:10.1371/journal.pone.0198926.

179. Dias, M.D.S.; Friche, A.D.L.; Mingoti, S.; Costa, D.D.S.; Andrade, A.D.S.; Freire, F.; Oliveira, V.D.; Caiaffa, W.T. Mortality from Homicides in Slums in the City of Belo Horizonte, Brazil: An Evaluation of the Impact of a Re-Urbanization Project. *International Journal of Environmental Research and Public Health* **2019**, *16*, 154. doi:10.3390/ijerph16010154.
180. Greif, M.J.; Dodoo, F.N.A. How community physical, structural, and social stressors relate to mental health in the urban slums of Accra, Ghana. *Health & Place* **2015**, *33*, 57–66. doi:10.1016/j.healthplace.2015.02.002.
181. Collishaw, S.; Gardner, F.; Aber, J.L.; Cluver, L. Predictors of Mental Health Resilience in Children who Have Been Parentally Bereaved by AIDS in Urban South Africa. *Journal of Abnormal Child Psychology* **2015**, *44*, 719–730. doi:10.1007/s10802-015-0068-x.
182. Nasreen, H.E.; Alam, M.A.; Edhborg, M. Prevalence and Associated Factors of Depressive Symptoms Among Disadvantaged Adolescents: Results from a Population-Based Study in Bangladesh. *Journal of Child and Adolescent Psychiatric Nursing* **2016**, *29*, 135–144. doi:10.1111/jcap.12150.
183. Williams, A.; Sarker, M.; Ferdous, S.T. Cultural Attitudes toward Postpartum Depression in Dhaka, Bangladesh. *Medical Anthropology* **2017**, *37*, 194–205. doi:10.1080/01459740.2017.1318875.
184. Panigrahi, A.; Das, S.C.; Sahoo, P. Adaptive functioning and its associated factors among girl children residing in slum areas of Bhubaneswar, India. *Journal of Paediatrics and Child Health* **2017**, *54*, 55–60. doi:10.1111/jpc.13666.
185. Liu, C.H.; Fink, G.; Brentani, H.; Brentani, A. An assessment of hair cortisol among postpartum Brazilian mothers and infants from a high-risk community in São Paulo: Intra-individual stability and association in mother-infant dyads. *Developmental Psychobiology* **2017**, *59*, 916–926. doi:10.1002/dev.21557.
186. Husain, N.; Zulqernain, F.; Carter, L.A.; Chaudhry, I.B.; Fatima, B.; Kiran, T.; Chaudhry, N.; Naeem, S.; Jafri, F.; Lunat, F.; Haq, S.U.; Husain, M.; Roberts, C.; Naeem, F.; Rahman, A. Treatment of maternal depression in urban slums of Karachi, Pakistan: A randomized controlled trial (RCT) of an integrated maternal psychological and early child development intervention. *Asian Journal of Psychiatry* **2017**, *29*, 63–70. doi:10.1016/j.ajp.2017.03.010.
187. Oluoch, P.; Orwa, J.; Lugalia, F.; Mutinda, D.; Gichangi, A.; Oundo, J.; Karama, M.; Nganga, Z.; Galbraith, J. Application of psychosocial models to Home-Based Testing and Counseling (HBTC) for increased uptake and household coverage in a large informal urban settlement in Kenya. *Pan African Medical Journal* **2017**, *27*. doi:10.11604/pamj.2017.27.285.10104.
188. Islam, S.; Nusrat, F.; Esha, S.; Mehrin, F.; Hilaly, A.; Shiraji, S.; Hasan, M.; Tofail, F.; Hamadani, J. How Does Family Structure Relate With Children's Language Development? A Cross Sectional Experience from Urban Slums in Dhaka. *Mymensingh medical journal: MMJ* **2017**, *26*, 775–782.
189. Culbreth, R.; Swahn, M.; Ndetei, D.; Ametewee, L.; Kasirye, R. Suicidal Ideation among Youth Living in the Slums of Kampala, Uganda. *International Journal of Environmental Research and Public Health* **2018**, *15*, 298. doi:10.3390/ijerph15020298.
190. Gibbs, A.; Dunkle, K.; Jewkes, R. Emotional and economic intimate partner violence as key drivers of depression and suicidal ideation: A cross-sectional study among young women in informal settlements in South Africa. *PLOS ONE* **2018**, *13*, e0194885. doi:10.1371/journal.pone.0194885.
191. Rani, D.; Singh, J.; Acharya, D.; Paudel, R.; Lee, K.; Singh, S. Household Food Insecurity and Mental Health Among Teenage Girls Living in Urban Slums in Varanasi, India: A Cross-Sectional Study. *International Journal of Environmental Research and Public Health* **2018**, *15*, 1585. doi:10.3390/ijerph15081585.
192. Ayyub, H.; Sarfraz, M.; Mir, K.; tus Salam, F. Association of antenatal depression and household food insecurity among pregnant women: a cross-sectional study from slums of Lahore. *Journal of Ayub Medical College Abbottabad* **2018**, *30*, 366–371.
193. Azad, R.; Fahmi, R.; Shrestha, S.; Joshi, H.; Hasan, M.; Khan, A.N.S.; Chowdhury, M.A.K.; Arifeen, S.E.; Billah, S.M. Prevalence and risk factors of postpartum depression within one year after birth in urban slums of Dhaka, Bangladesh. *PLOS ONE* **2019**, *14*, e0215735. doi:10.1371/journal.pone.0215735.
194. Murthy, N.; Chandrasekharan, S.; Prakash, M.P.; Kaonga, N.N.; Peter, J.; Ganju, A.; Mechael, P.N. The Impact of an mHealth Voice Message Service (mMitra) on Infant Care Knowledge, and Practices Among Low-Income Women in India: Findings from a Pseudo-Randomized Controlled Trial. *Maternal and Child Health Journal* **2019**, *23*, 1658–1669. doi:10.1007/s10995-019-02805-5.

195. Khopkar, S.; Kulathinal, S.; Virtanen, S.M.; Säävälä, M. Age at menarche and diet among adolescents in slums of Nashik, India. *International Journal of Adolescent Medicine and Health* **2015**, *27*, 451–456. doi:10.1515/ijamh-2014-0056.
196. Madiba, S.; Chelule, P.K.; Mokgatle, M.M. Attending Informal Preschools and Daycare Centers Is a Risk Factor for Underweight, Stunting and Wasting in Children under the Age of Five Years in Underprivileged Communities in South Africa. *International Journal of Environmental Research and Public Health* **2019**, *16*, 2589. doi:10.3390/ijerph16142589.
197. Florêncio, T.M.M.T.; Bueno, N.B.; Clemente, A.P.G.; Albuquerque, F.C.A.; Britto, R.P.A.; Ferriolli, E.; Sawaya, A.L. Weight gain and reduced energy expenditure in low-income Brazilian women living in slums: a 4-year follow-up study. *British Journal of Nutrition* **2015**, *114*, 462–471. doi:10.1017/s0007114515001816.
198. Savanur, M.S.; Ghugre, P.S. Magnitude of undernutrition in children aged 2 to 4 years using CIAF and conventional indices in the slums of Mumbai city. *Journal of Health, Population and Nutrition* **2015**, *33*. doi:10.1186/s41043-015-0017-x.
199. Sahariah, S.A.; Potdar, R.D.; Gandhi, M.; Kehoe, S.H.; Brown, N.; Sane, H.; Coakley, P.J.; Marley-Zagar, E.; Chopra, H.; Shivshankaran, D.; Cox, V.A.; Jackson, A.A.; Margetts, B.M.; Fall, C.H. A Daily Snack Containing Leafy Green Vegetables, Fruit, and Milk before and during Pregnancy Prevents Gestational Diabetes in a Randomized, Controlled Trial in Mumbai, India. *The Journal of Nutrition* **2016**, *146*, 1453S–1460S. doi:10.3945/jn.115.223461.
200. Kabir, A.; Maitrot, M.R.L. Factors influencing feeding practices of extreme poor infants and young children in families of working mothers in Dhaka slums: A qualitative study. *PLOS ONE* **2017**, *12*, e0172119. doi:10.1371/journal.pone.0172119.
201. Lawande, A.; Gravio, C.D.; Potdar, R.D.; Sahariah, S.A.; Gandhi, M.; Chopra, H.; Sane, H.; Kehoe, S.H.; Marley-Zagar, E.; Margetts, B.M.; Jackson, A.A.; Fall, C.H.D. Effect of a micronutrient-rich snack taken preconceptionally and throughout pregnancy on ultrasound measures of fetal growth: The Mumbai Maternal Nutrition Project (MMNP). *Maternal & Child Nutrition* **2017**, *14*, e12441. doi:10.1111/mcn.12441.
202. Malshe, S.D.; Udipi, S.A. Waist-to-Height Ratio in Indian Women: Comparison With Traditional Indices of Obesity, Association With Inflammatory Biomarkers and Lipid Profile. *Asia Pacific Journal of Public Health* **2017**, *29*, 411–421. doi:10.1177/1010539517717509.
203. Jeyakumar, A.; Ghugre, P. Is lack of breakfast contributing to nutrient deficits and poor nutritional indicators among adolescent girls? *Nutrition and Health* **2017**, *23*, 177–184. doi:10.1177/0260106017721074.
204. Mehta, S.; Finkelstein, J.L.; Venkatramanan, S.; Huey, S.L.; Udipi, S.A.; Ghugre, P.; Ruth, C.; Canfield, R.L.; Kurpad, A.V.; Potdar, R.D.; Haas, J.D. Effect of iron and zinc-biofortified pearl millet consumption on growth and immune competence in children aged 12-18 months in India: study protocol for a randomised controlled trial. *BMJ Open* **2017**, *7*, e017631. doi:10.1136/bmjopen-2017-017631.
205. Mostafa, I.; Naila, N.N.; Mahfuz, M.; Roy, M.; Faruque, A.S.; Ahmed, T. Children living in the slums of Bangladesh face risks from unsafe food and water and stunted growth is common. *Acta Paediatrica* **2018**, *107*, 1230–1239. doi:10.1111/apa.14281.
206. More, N.S.; Waingankar, A.; Ramani, S.; Chanani, S.; Souza, V.D.; Pantvaidya, S.; Fernandez, A.; Jayaraman, A. Community-Based Management of Acute Malnutrition to Reduce Wasting in Urban Informal Settlements of Mumbai, India: A Mixed-Methods Evaluation. *Global Health: Science and Practice* **2018**, *6*, 103–127. doi:10.9745/ghsp-d-17-00182.
207. Goudet, S.; Jayaraman, A.; Chanani, S.; Osrin, D.; Devleesschauwer, B.; Bogin, B.; Madise, N.; Griffiths, P. Cost effectiveness of a community based prevention and treatment of acute malnutrition programme in Mumbai slums, India. *PLOS ONE* **2018**, *13*, e0205688. doi:10.1371/journal.pone.0205688.
208. Jadhav, A.R.; Karnik, P.; Fernandes, L.; Fernandes, S.; Shah, N.; Manglani, M. Indigenously Prepared Ready-to-use Therapeutic Food (RUTF) in Children with Severe Acute Malnutrition. *Indian Pediatrics* **2019**, *56*, 287–293. doi:10.1007/s13312-019-1516-4.
209. Vita, M.V.D.; Scolfaro, C.; Santini, B.; Lezo, A.; Gobbi, F.; Buonfrate, D.; Kimani-Murage, E.W.; Macharia, T.; Wanjohi, M.; Rovarini, J.M.; Morino, G. Malnutrition, morbidity and infection in the informal settlements of Nairobi, Kenya: an epidemiological study. *Italian Journal of Pediatrics* **2019**, *45*. doi:10.1186/s13052-019-0607-0.
210. Faye, C.M.; Fonn, S.; Levin, J. Factors associated with recovery from stunting among under-five children in two Nairobi informal settlements. *PLOS ONE* **2019**, *14*, e0215488. doi:10.1371/journal.pone.0215488.

211. Ndirangu, G.; Gichangi, A.; Kanyuuru, L.; Otai, J.; Mulindi, R.; Lynam, P.; Koskei, N.; Tappis, H.; Archer, L. Using Young Mothers' Clubs to Improve Knowledge of Postpartum Hemorrhage and Family Planning in Informal Settlements in Nairobi, Kenya. *Journal of Community Health* **2015**, *40*, 692–698. doi:10.1007/s10900-014-9986-8.
212. Kamndaya, M.; Kazembe, L.N.; Vearey, J.; Kabiru, C.W.; Thomas, L. Material deprivation and unemployment affect coercive sex among young people in the urban slums of Blantyre, Malawi: A multi-level approach. *Health & Place* **2015**, *33*, 90–100. doi:10.1016/j.healthplace.2015.03.001.
213. Ghosh-Jerath, S.; Devasenapathy, N.; Singh, A.; Shankar, A.; Zodpey, S. Ante natal care (ANC) utilization, dietary practices and nutritional outcomes in pregnant and recently delivered women in urban slums of Delhi, India: an exploratory cross-sectional study. *Reproductive Health* **2015**, *12*. doi:10.1186/s12978-015-0008-9.
214. Beguy, D.; Mberu, B. Patterns of fertility preferences and contraceptive behaviour over time: change and continuities among the urban poor in Nairobi, Kenya. *Culture, Health & Sexuality* **2015**, *17*, 1074–1089. doi:10.1080/13691058.2015.1038731.
215. Khan, A.A.; Varan, A.K.; Esteves-Jaramillo, A.; Siddiqui, M.; Sultana, S.; Ali, A.S.; Zaidi, A.K.; Omer, S.B. Influenza vaccine acceptance among pregnant women in urban slum areas, Karachi, Pakistan. *Vaccine* **2015**, *33*, 5103–5109. doi:10.1016/j.vaccine.2015.08.014.
216. Mahmud, I.; Chowdhury, S.; Siddiqi, B.A.; Theobald, S.; Ormel, H.; Biswas, S.; Jahangir, Y.T.; Sarker, M.; Rashid, S.F. Exploring the context in which different close-to-community sexual and reproductive health service providers operate in Bangladesh: a qualitative study. *Human Resources for Health* **2015**, *13*. doi:10.1186/s12960-015-0045-z.
217. Devasenapathy, N.; Jerath, S.G.; Allen, E.; Sharma, S.; Shankar, A.H.; Zodpey, S. Reproductive healthcare utilization in urban poor settlements of Delhi: Baseline survey of ANCHUL (Ante Natal and Child Health care in Urban Slums) project. *BMC Pregnancy and Childbirth* **2015**, *15*. doi:10.1186/s12884-015-0635-8.
218. Alcock, G.; Das, S.; More, N.S.; Hate, K.; More, S.; Pantvaidya, S.; Osrin, D.; Houweling, T.A. Examining inequalities in uptake of maternal health care and choice of provider in underserved urban areas of Mumbai, India: a mixed methods study. *BMC Pregnancy and Childbirth* **2015**, *15*. doi:10.1186/s12884-015-0661-6.
219. Mumah, J.N.; Machiyama, K.; Mutua, M.; Kabiru, C.W.; Cleland, J. Contraceptive Adoption, Discontinuation, and Switching among Postpartum Women in Nairobi s Urban Slums. *Studies in Family Planning* **2015**, *46*, 369–386. doi:10.1111/j.1728-4465.2015.00038.x.
220. Kimani-Murage, E.W.; Norris, S.A.; Mutua, M.K.; Wekesah, F.; Wanjohi, M.; Muhia, N.; Muriuki, P.; Egondi, T.; Kyobutungi, C.; Ezeh, A.C.; Musoke, R.N.; McGarvey, S.T.; Madise, N.J.; Griffiths, P.L. Potential effectiveness of Community Health Strategy to promote exclusive breastfeeding in urban poor settings in Nairobi, Kenya: a quasi-experimental study. *Journal of Developmental Origins of Health and Disease* **2015**, *7*, 172–184. doi:10.1017/s2040174415007941.
221. Diamond-Smith, N.; Sudhinaraset, M.; Melo, J.; Murthy, N. The relationship between women s experiences of mistreatment at facilities during childbirth, types of support received and person providing the support in Lucknow, India. *Midwifery* **2016**, *40*, 114–123. doi:10.1016/j.midw.2016.06.014.
222. Sarnquist, C.; Sinclair, J.; Mboya, B.O.; Langat, N.; Paiva, L.; Halpern-Felsher, B.; Golden, N.H.; Maldonado, Y.A.; Baiocchi, M.T. Evidence That Classroom-Based Behavioral Interventions Reduce Pregnancy-Related School Dropout Among Nairobi Adolescents. *Health Education & Behavior* **2016**, *44*, 297–303. doi:10.1177/1090198116657777.
223. Baiocchi, M.; Omondi, B.; Langat, N.; Boothroyd, D.B.; Sinclair, J.; Pavia, L.; Mulinge, M.; Githua, O.; Golden, N.H.; Sarnquist, C. A Behavior-Based Intervention That Prevents Sexual Assault: the Results of a Matched-Pairs, Cluster-Randomized Study in Nairobi, Kenya. *Prevention Science* **2016**, *18*, 818–827. doi:10.1007/s11121-016-0701-0.
224. Jolly, S.P.; Rahman, M.; Afsana, K.; Yunus, F.M.; Chowdhury, A.M.R. Evaluation of Maternal Health Service Indicators in Urban Slum of Bangladesh. *PLOS ONE* **2016**, *11*, e0162825. doi:10.1371/journal.pone.0162825.
225. Cohen, J.; Golub, G.; Kruk, M.E.; McConnell, M. Do active patients seek higher quality prenatal care?: A panel data analysis from Nairobi, Kenya. *Preventive Medicine* **2016**, *92*, 74–81. doi:10.1016/j.ypmed.2016.09.014.

226. Renzaho, A.M.N.; Kamara, J.K.; Georgeou, N.; Kamanga, G. Sexual, Reproductive Health Needs, and Rights of Young People in Slum Areas of Kampala, Uganda: A Cross Sectional Study. *PLOS ONE* **2017**, *12*, e0169721. doi:10.1371/journal.pone.0169721.
227. Sahu, K.S.; Bharati, B.; others. Out-of-Pocket health expenditure and sources of financing for delivery, postpartum, and neonatal health in urban slums of Bhubaneswar, Odisha, India. *Indian journal of public health* **2017**, *61*, 67.
228. Bello, B.; Moultrie, H.; Somji, A.; Chersich, M.F.; Watts, C.; Delany-Moretlwe, S. Alcohol use and sexual risk behaviour among men and women in inner-city Johannesburg, South Africa. *BMC Public Health* **2017**, *17*. doi:10.1186/s12889-017-4350-4.
229. Diamond-Smith, N.; Treleaven, E.; Murthy, N.; Sudhinaraset, M. Women's empowerment and experiences of mistreatment during childbirth in facilities in Lucknow, India: results from a cross-sectional study. *BMC Pregnancy and Childbirth* **2017**, *17*. doi:10.1186/s12884-017-1501-7.
230. Hill, L.; Moody, J.; Gottfredson, N.; Kajula, L.; Pence, B.; Go, V.; Maman, S. Peer norms moderate the association between mental health and sexual risk behaviors among young men living in Dar es Salaam, Tanzania. *Social Science & Medicine* **2018**, *196*, 77–85. doi:10.1016/j.socscimed.2017.10.030.
231. Atusiimire, L.B.; Waiswa, P.; Atuyambe, L.; Nankabirwa, V.; Okuga, M. Determinants of facility based-deliveries among urban slum dwellers of Kampala, Uganda. *PLOS ONE* **2019**, *14*, e0214995. doi:10.1371/journal.pone.0214995.
232. Nahar, S.; Akhter, S.; Ahamed, F.; Akhtar, K.; Noor, F. Study on Clinical Presentation and Outcome of Septic Abortion and Its Relationship with Person Inducing Abortion. *Mymensingh medical journal: MMJ* **2017**, *26*, 699–704.
233. Chanani, S.; Waingankar, A.; More, N.S.; Pantvaidya, S.; Fernandez, A.; Jayaraman, A. Participation of pregnant women in a community-based nutrition program in Mumbais informal settlements: Effect on exclusive breastfeeding practices. *PLOS ONE* **2018**, *13*, e0195619. doi:10.1371/journal.pone.0195619.
234. Ara, G.; Khanam, M.; Papri, N.; Nahar, B.; Haque, M.A.; Kabir, I.; Dibley, M.J. Peer counselling improves breastfeeding practices: A cluster randomized controlled trial in urban Bangladesh. *Maternal & Child Nutrition* **2018**, *14*. doi:10.1111/mcn.12605.
235. Kusuma, Y.S.; Kaushal, S.; Garg, R.; Babu, B.V. Birth preparedness and determinants of birth place among migrants living in slums and slum-like pockets in Delhi, India. *Sexual & Reproductive Healthcare* **2018**, *16*, 160–166. doi:10.1016/j.srhc.2018.04.004.
236. Oyugi, B.; Kioko, U.; Kaboro, S.M.; Okumu, C.; Ogola-Munene, S.; Kalsi, S.; Thiani, S.; Gikonyo, S.; Korir, J.; Baltazar, B.; Ranji, M. A facility-based study of women' satisfaction and perceived quality of reproductive and maternal health services in the Kenya output-based approach voucher program. *BMC Pregnancy and Childbirth* **2018**, *18*. doi:10.1186/s12884-018-1940-9.
237. Mumah, J.N.; Casterline, J.B.; Machiyama, K.; Wamukoya, M.; Kabiru, C.W.; Cleland, J. Method-Specific Attributes that Influence Choice of Future Contraception Among Married Women in Nairobis Informal Settlements. *Studies in Family Planning* **2018**, *49*, 279–292. doi:10.1111/sifp.12070.
238. Jeyashree, K.; Kathirvel, S.; Trusty, K.; Singh, A. Socio-demographic factors affecting the choice of place of childbirth among migrant and native women - A case control study from Chandigarh, India. *Sexual & Reproductive Healthcare* **2018**, *17*, 81–85. doi:10.1016/j.srhc.2018.07.006.
239. Kaur, M.; Gupta, M.; Purayil, V.P.; Rana, M.; Chakrapani, V. Contribution of social factors to maternal deaths in urban India: Use of care pathway and delay models. *PLOS ONE* **2018**, *13*, e0203209. doi:10.1371/journal.pone.0203209.
240. Owiti, A.; Oyugi, J.; Essink, D. Utilization of Kenya's free maternal health services among women living in Kibera slums: a cross-sectional study. *Pan African Medical Journal* **2018**, *30*. doi:10.11604/pamj.2018.30.86.15151.
241. Atinga, R.A.; Abiiro, G.A.; Kuganab-Lem, R.B. Factors influencing the decision to drop out of health insurance enrolment among urban slum dwellers in Ghana. *Tropical Medicine & International Health* **2014**, *20*, 312–321. doi:10.1111/tmi.12433.
242. Beguy, D.; ata, P.E.; Mberu, B.; Oduor, C.; Wamukoya, M.; Nganyi, B.; Ezeh, A. Health & Demographic Surveillance System Profile: The Nairobi Urban Health and Demographic Surveillance System (NUHDSS). *International Journal of Epidemiology* **2015**, *44*, 462–471. doi:10.1093/ije/dyu251.

243. O'Keefe, M.; Messmer, U.; Lüthi, C.; Tobias, R. Slum inhabitants' perceptions and decision-making processes related to an innovative sanitation service: evaluating the Blue Diversion Toilet in Kampala (Uganda). *International Journal of Environmental Health Research* **2015**, *25*, 670–684. doi:10.1080/09603123.2015.1007842.
244. Sharma, V.; Singh, A.; Sharma, V. Provider's and User's Perspective about Immunization Coverage among Migratory and Non-migratory Population in Slums and Construction Sites of Chandigarh. *Journal of Urban Health* **2015**, *92*, 304–312. doi:10.1007/s11524-015-9939-2.
245. Egondi, T.; Kyobutungi, C.; Rocklöv, J. Temperature Variation and Heat Wave and Cold Spell Impacts on Years of Life Lost Among the Urban Poor Population of Nairobi, Kenya. *International Journal of Environmental Research and Public Health* **2015**, *12*, 2735–2748. doi:10.3390/ijerph120302735.
246. Mberu, B.; Wamukoya, M.; Oti, S.; Kyobutungi, C. Trends in Causes of Adult Deaths among the Urban Poor: Evidence from Nairobi Urban Health and Demographic Surveillance System, 2003-2012. *Journal of Urban Health* **2015**, *92*, 422–445. doi:10.1007/s11524-015-9943-6.
247. Adams, A.M.; Islam, R.; Ahmed, T. Who serves the urban poor? A geospatial and descriptive analysis of health services in slum settlements in Dhaka, Bangladesh. *Health Policy and Planning* **2015**, *30*, i32–i45. doi:10.1093/heapol/czu094.
248. Egondi, T.; Oyolola, M.; Mutua, M.K.; Elung'ata, P. Determinants of immunization inequality among urban poor children: evidence from Nairobi's informal settlements. *International Journal for Equity in Health* **2015**, *14*. doi:10.1186/s12939-015-0154-2.
249. Khabala, K.B.; Edwards, J.K.; Baruani, B.; Sirengo, M.; Musembi, P.; Kosgei, R.J.; Walter, K.; Kibachio, J.M.; Tondoi, M.; Ritter, H.; Wilkinson, E.; Reid, T. Medication Adherence Clubs: a potential solution to managing large numbers of stable patients with multiple chronic diseases in informal settlements. *Tropical Medicine & International Health* **2015**, *20*, 1265–1270. doi:10.1111/tmi.12539.
250. Mahapatra, T.; Mahapatra, S.; Pal, D.; Saha, J.; Lopez, A.; Ali, M.; Bannerjee, B.; Manna, B.; Sur, D.; Bhattacharya, S.; Kanungo, S. Trials and tribulations of conducting interventional studies in urban slums of a developing country: Experiences from Kolkata, India. *Human Vaccines & Immunotherapeutics* **2015**, *12*, 182–186. doi:10.1080/21645515.2015.1066052.
251. Goel, N.K.; Pathak, R.; Gulati, S.; Balakrishnan, S.; Singh, N.; Singh, H. Surveillance of bacteriological quality of drinking water in Chandigarh, northern India. *Journal of Water and Health* **2015**, *13*, 931–938. doi:10.2166/wh.2015.132.
252. Tumwebaze, I.K.; Mosler, H.J. Effectiveness of group discussions and commitment in improving cleaning behaviour of shared sanitation users in Kampala, Uganda slums. *Social Science & Medicine* **2015**, *147*, 72–79. doi:10.1016/j.socscimed.2015.10.059.
253. de Lima Friche, A.A.; de Salles Dias, M.A.; dos Reis, P.B.; Dias, C.S.; Caiaffa, W.T. Urban upgrading and its impact on health: a "quasi-experimental" mixed-methods study protocol for the BH-Viva Project. *Cadernos de Saúde Pública* **2015**, *31*, 51–64. doi:10.1590/0102-311x00079715.
254. Nekoei-Moghadam, M.; Heidari, N.; Amiresmaeili, M.; Heidarijamebozorgi, M. Prioritizing the health problems of slum residents using social determinants of health: A case study in a developing country. *The International Journal of Health Planning and Management* **2019**, *34*. doi:10.1002/hpm.2800.
255. McDowell, A.; Pai, M. Alternative medicine: an ethnographic study of how practitioners of Indian medical systems manage TB in Mumbai. *Transactions of The Royal Society of Tropical Medicine and Hygiene* **2016**, *110*, 192–198. doi:10.1093/trstmh/trw009.
256. Delavallade, C. Quality Health Care and Willingness to Pay for Health Insurance Retention: A Randomized Experiment in Kolkata Slums. *Health Economics* **2016**, *26*, 619–638. doi:10.1002/hec.3337.
257. Kwiringira, J.; Atekyereza, P.; Niwagaba, C.; Kabumbuli, R.; Rwabukwali, C.; Kulabako, R.; Günther, I. Seasonal variations and shared latrine cleaning practices in the slums of Kampala city, Uganda. *BMC Public Health* **2016**, *16*. doi:10.1186/s12889-016-3036-7.
258. Morrow, M.; Armstrong, G.; Dayal, P.; Kermode, M. Documenting a long-term development model in the slums of Delhi. *BMC International Health and Human Rights* **2016**, *16*. doi:10.1186/s12914-016-0088-9.
259. Mitchell, C.; Chege, F.; Maina, L.; Rothman, M. Beyond engagement in working with children in eight Nairobi slums to address safety, security, and housing: Digital tools for policy and community dialogue. *Global Public Health* **2016**, *11*, 651–665. doi:10.1080/17441692.2016.1165720.

260. Bennett, R.; Chepngeno-Langat, G.; Evandrou, M.; Falkingham, J. Gender differentials and old age survival in the Nairobi slums, Kenya. *Social Science & Medicine* **2016**, *163*, 107–116. doi:10.1016/j.socscimed.2016.07.002.
261. Awunyo-Akaba, Y.; Awunyo-Akaba, J.; Gyapong, M.; Senah, K.; Konradsen, F.; Rheinländer, T. Sanitation investments in Ghana: An ethnographic investigation of the role of tenure security, land ownership and livelihoods. *BMC Public Health* **2016**, *16*. doi:10.1186/s12889-016-3283-7.
262. Beletsky, L.; Arredondo, J.; Werb, D.; Vera, A.; Abramovitz, D.; Amon, J.J.; Brouwer, K.C.; Strathdee, S.A.; Gaines, T.L. Utilization of Google enterprise tools to georeference survey data among hard-to-reach groups: strategic application in international settings. *International Journal of Health Geographics* **2016**, *15*. doi:10.1186/s12942-016-0053-9.
263. Harrell, M.B.; Arora, M.; Bassi, S.; Gupta, V.K.; Perry, C.L.; Reddy, K.S. Reducing tobacco use among low socio-economic status youth in Delhi, India: outcomes from project ACTIVITY, a cluster randomized trial. *Health Education Research* **2016**, *31*, 624–638. doi:10.1093/her/cyw039.
264. Lungu, E.A.; Biesma, R.; Chirwa, M.; Darker, C. Healthcare seeking practices and barriers to accessing under-five child health services in urban slums in Malawi: a qualitative study. *BMC Health Services Research* **2016**, *16*. doi:10.1186/s12913-016-1678-x.
265. Lee, G.O.; Olortegui, M.P.; Salmón-Mulanovich, G.; Yori, P.P.; Kosek, M. Early child health in an informal settlement in the Peruvian Amazon. *BMC International Health and Human Rights* **2016**, *16*. doi:10.1186/s12914-016-0099-6.
266. Tobias, R.; OKeefe, M.; Künzle, R.; Gebauer, H.; Gründl, H.; Morgenroth, E.; Pronk, W.; Larsen, T.A. Early testing of new sanitation technology for urban slums: The case of the Blue Diversion Toilet. *Science of The Total Environment* **2017**, *576*, 264–272. doi:10.1016/j.scitotenv.2016.10.057.
267. Sengupta, P.; Benjamin, A.I.; Myles, P.R.; Babu, B.V. Evaluation of a community-based intervention to improve routine childhood vaccination uptake among migrants in urban slums of Ludhiana, India. *Journal of Public Health* **2016**, *39*, 805–812. doi:10.1093/pubmed/fdw131.
268. Bohnert, K.; Chard, A.; Mwaki, A.; Kirby, A.; Muga, R.; Nagel, C.; Thomas, E.; Freeman, M. Comparing Sanitation Delivery Modalities in Urban Informal Settlement Schools: A Randomized Trial in Nairobi, Kenya. *International Journal of Environmental Research and Public Health* **2016**, *13*, 1189. doi:10.3390/ijerph13121189.
269. Mishra, S.; Kusuma, Y.S.; Babu, B.V. Treatment-seeking and out-of-pocket expenditure on childhood illness in a migrant tribal community in Bhubaneswar, Odisha State, India. *Paediatrics and International Child Health* **2016**, *37*, 181–187. doi:10.1080/20469047.2016.1245031.
270. Mishra, S.; Kusuma, Y.S.; Babu, B.V. Mother's Recognition of and Treatment Triggers for Common Childhood Illnesses among Migrant Santal Tribe Living in Bhubaneswar, Odisha, India. *Journal of Tropical Pediatrics* **2016**, p. fmw092. doi:10.1093/tropej/fmw092.
271. Hutain, J.; Perry, H.B.; Koffi, A.K.; Christensen, M.; OConnor, E.C.; Jabbi, S.M.B.B.; Samba, T.T.; Kaiser, R. Engaging communities in collecting and using results from verbal autopsies for child deaths: an example from urban slums in Freetown, Sierra Leone. *Journal of Global Health* **2019**, *9*. doi:10.7189/jogh.09.010419.
272. O'Connor, E.C.; Hutain, J.; Christensen, M.; Kamara, M.S.; Conteh, A.; Sarriot, E.; Samba, T.T.; Perry, H.B. Piloting a participatory, community-based health information system for strengthening community-based health services: findings of a cluster-randomized controlled trial in the slums of Freetown, Sierra Leone. *Journal of Global Health* **2019**, *9*. doi:10.7189/jogh.09.010418.
273. Simiyu, S.; Swilling, M.; Rheingans, R.; Cairncross, S. Estimating the Cost and Payment for Sanitation in the Informal Settlements of Kisumu, Kenya: A Cross Sectional Study. *International Journal of Environmental Research and Public Health* **2017**, *14*, 49. doi:10.3390/ijerph14010049.
274. Simiyu, S.; Swilling, M.; Cairncross, S.; Rheingans, R. Determinants of quality of shared sanitation facilities in informal settlements: case study of Kisumu, Kenya. *BMC Public Health* **2017**, *17*. doi:10.1186/s12889-016-4009-6.
275. Nekoei-Moghadam, M.; Heidari, N.; Amiresmaeili, M.; Heidarijamebozorgi, M. Identifying the health problems of slum residents using social determinants of health: Kerman, Iran. *The International Journal of Health Planning and Management* **2019**, *34*. doi:10.1002/hpm.2755.
276. More, N.S.; Das, S.; Bapat, U.; Alcock, G.; Manjrekar, S.; Kamble, V.; Sawant, R.; Shende, S.; Daruwalla, N.; Pantvaidya, S.; Osrin, D. Community resource centres to improve the health of women and children

in informal settlements in Mumbai: a cluster-randomised, controlled trial. *The Lancet Global Health* **2017**, *5*, e335–e349. doi:10.1016/s2214-109x(16)30363-1.

277. Ngole-Jeme, V.M.; Fantke, P. Ecological and human health risks associated with abandoned gold mine tailings contaminated soil. *PLOS ONE* **2017**, *12*, e0172517. doi:10.1371/journal.pone.0172517.

278. Sharma, N.; Anand, T.; Grover, S.; Kumar, A.; Singh, M.M.; Ingle, G.K. Awareness About Anti-Smoking Related Laws and Legislation Among General Population in Slums of Delhi, India. *Nicotine & Tobacco Research* **2017**, *20*, 643–648. doi:10.1093/ntr/ntx098.

279. Alam, M.U.; Winch, P.J.; Saxton, R.E.; Nizame, F.A.; Yeasmin, F.; Norman, G.; Masud, A.A.; Begum, F.; Rahman, M.; Hossain, K.; Layden, A.; Unicomb, L.; Luby, S.P. Behaviour change intervention to improve shared toilet maintenance and cleanliness in urban slums of Dhaka: a cluster-randomised controlled trial. *Tropical Medicine & International Health* **2017**, *22*, 1000–1011. doi:10.1111/tmi.12902.

280. George, M.; Pant, S.; Devasenapathy, N.; Ghosh-Jerath, S.; Zodpey, S. Motivating and demotivating factors for community health workers: A qualitative study in urban slums of Delhi, India. *WHO South-East Asia Journal of Public Health* **2017**, *6*, 82. doi:10.4103/2224-3151.206170.

281. Gudda, F.O.; Moturi, W.N.; Oduor, O.S.; Muchiri, E.W.; Ensink, J. Pit latrine fill-up rates: variation determinants and public health implications in informal settlements, Nakuru-Kenya. *BMC Public Health* **2019**, *19*. doi:10.1186/s12889-019-6403-3.

282. Saxton, R.E.; Yeasmin, F.; Alam, M.U.; Al-Masud, A.; Dutta, N.C.; Yeasmin, D.; Luby, S.P.; Unicomb, L.; Winch, P.J. If I do not have enough water, then how could I bring additional water for toilet cleaning?! Addressing water scarcity to promote hygienic use of shared toilets in Dhaka, Bangladesh. *Tropical Medicine & International Health* **2017**, *22*, 1099–1111. doi:10.1111/tmi.12914.

283. Simiyu, S.; Swilling, M.; Cairncross, S. Decision-making on shared sanitation in the informal settlements of Kisumu, Kenya. *International Journal of Environmental Health Research* **2017**, *27*, 377–393. doi:10.1080/09603123.2017.1350261.

284. Kleczka, B.; Musiega, A.; Rabut, G.; Wekesa, P.; Mwaniki, P.; Marx, M.; Kumar, P. Rubber stamp templates for improving clinical documentation: A paper-based, m-Health approach for quality improvement in low-resource settings. *International Journal of Medical Informatics* **2018**, *114*, 121–129. doi:10.1016/j.ijmedinf.2017.10.014.

285. Bhattacherjee, S.; Bhar, D.; Mukherjee, A.; Sarkar, T.; Dasgupta, S. Utilization of safe drinking water and sanitary facilities in slum households of Siliguri, West Bengal. *Indian Journal of Public Health* **2017**, *61*, 248. doi:10.4103/ijph.ijph_345_16.

286. Lungu, E.A.; Obse, A.G.; Darker, C.; Biesma, R. What influences where they seek care? Caregivers' preferences for under-five child healthcare services in urban slums of Malawi: A discrete choice experiment. *PLOS ONE* **2018**, *13*, e0189940. doi:10.1371/journal.pone.0189940.

287. Abdi, S.; Wadugodapitiya, A.; Bedaf, S.; George, C.E.; Norman, G.; Hawley, M.; de Witte, L. Identification of priority health conditions for field-based screening in urban slums in Bangalore, India. *BMC Public Health* **2018**, *18*. doi:10.1186/s12889-018-5194-2.

288. Hunter, B.M. Brokerage in commercialised healthcare systems: A conceptual framework and empirical evidence from Uttar Pradesh. *Social Science & Medicine* **2018**, *202*, 128–135. doi:10.1016/j.socscimed.2018.03.004.

289. Makadzange, K.; Radebe, Z.; Maseko, N.; Lukhele, V.; Masuku, S.; Fakudze, G.; Mengestu, T.K.; Prasad, A. Implementation of Urban Health Equity Assessment and Response Tool: a Case of Matsapha, Swaziland. *Journal of Urban Health* **2018**, *95*, 672–681. doi:10.1007/s11524-018-0241-y.

290. Helgegren, I.; Rauch, S.; Cossio, C.; Landaeta, G.; McConville, J. Importance of triggers and veto-barriers for the implementation of sanitation in informal peri-urban settlements - The case of Cochabamba, Bolivia. *PLOS ONE* **2018**, *13*, e0193613. doi:10.1371/journal.pone.0193613.

291. Aleemi, A.R.; Khaliqui, H.; Faisal, A. Challenges and Patterns of Seeking Primary Health Care in Slums of Karachi: A Disaster Lurking in Urban Shadows. *Asia Pacific Journal of Public Health* **2018**, *30*, 479–490. doi:10.1177/1010539518772132.

292. Černauskas, V.; Angeli, F.; Jaiswal, A.K.; Pavlova, M. Underlying determinants of health provider choice in urban slums: results from a discrete choice experiment in Ahmedabad, India. *BMC Health Services Research* **2018**, *18*. doi:10.1186/s12913-018-3264-x.

293. Das, M.; Elsey, H.; Shawon, R.A.; Hicks, J.; Ferdoush, J.; Huque, R.; Fieroze, F.; Nasreen, S.; Wallace, H.; Mashreky, S.R. Protocol to develop sustainable day care for children aged 1-4 years in disadvantaged urban communities in Dhaka, Bangladesh. *BMJ Open* **2018**, *8*, e024101. doi:10.1136/bmjopen-2018-024101.
294. Gibbs, A.; Jewkes, R.; Willan, S.; Washington, L. Associations between poverty, mental health and substance use, gender power, and intimate partner violence amongst young (18-30) women and men in urban informal settlements in South Africa: A cross-sectional study and structural equation model. *PLOS ONE* **2018**, *13*, e0204956. doi:10.1371/journal.pone.0204956.
295. Mukiira, C.; Ibisomi, L. Health care seeking practices of caregivers of children under 5 with diarrhea in two informal settlements in Nairobi, Kenya. *Journal of Child Health Care* **2013**, *19*, 254–264. doi:10.1177/1367493513508231.
296. Mutua, M.K.; Ochako, R.; Ettarh, R.; Ravn, H.; Echoka, E.; Mwaniki, P. Effects of low birth weight on time to BCG vaccination in an urban poor settlement in Nairobi, Kenya: an observational cohort study. *BMC Pediatrics* **2015**, *15*. doi:10.1186/s12887-015-0360-5.
297. Kerubo, G.; Khamadi, S.; Okoth, V.; Madise, N.; Ezeh, A.; Abdalla, Z.; Mwau, M. Hepatitis B, Hepatitis C and HIV-1 Coinfection in Two Informal Urban Settlements in Nairobi, Kenya. *PLOS ONE* **2015**, *10*, e0129247. doi:10.1371/journal.pone.0129247.
298. Muhula, S.O.; Peter, M.; Sibhatu, B.; Meshack, N.; Lennie, K. Effects of highly active antiretroviral therapy on the survival of hiv-infected adult patients in urban slums of Kenya. *Pan African Medical Journal* **2015**, *20*. doi:10.11604/pamj.2015.20.63.4865.
299. Moto, J.N.; Maingi, J.M.; Nyamache, A.K. Prevalence of Tinea capitis in school going children from Mathare, informal settlement in Nairobi, Kenya. *BMC Research Notes* **2015**, *8*. doi:10.1186/s13104-015-1240-7.
300. Kikuti, M.; Cunha, G.M.; Paploski, I.A.D.; Kasper, A.M.; Silva, M.M.O.; Tavares, A.S.; Cruz, J.S.; Queiroz, T.L.; Rodrigues, M.S.; Santana, P.M.; Lima, H.C.A.V.; Calcagno, J.; Takahashi, D.; Gonçalves, A.H.O.; Araújo, J.M.G.; Gauthier, K.; Diuk-Wasser, M.A.; Kitron, U.; Ko, A.I.; Reis, M.G.; Ribeiro, G.S. Spatial Distribution of Dengue in a Brazilian Urban Slum Setting: Role of Socioeconomic Gradient in Disease Risk. *PLOS Neglected Tropical Diseases* **2015**, *9*, e0003937. doi:10.1371/journal.pntd.0003937.
301. Blanton, E.; Wilhelm, N.; Reilly, C.O.; Muhonja, E.; Karoki, S.; Ope, M.; Langat, D.; Omolo, J.; Wamola, N.; Oundo, J.; Hoekstra, R.; Ayers, T.; Cock, K.D.; Breiman, R.; Mintz, E.; Lantagne, D. A rapid assessment of drinking water quality in informal settlements after a cholera outbreak in Nairobi, Kenya. *Journal of Water and Health* **2015**, *13*, 714–725. doi:10.2166/wh.2014.173.
302. Njuguna, H.N.; Montgomery, J.M.; Cosmas, L.; Wamola, N.; Oundo, J.O.; Desai, M.; Buff, A.M.; Breiman, R.F. Malaria Parasitemia Among Febrile Patients Seeking Clinical Care at an Outpatient Health Facility in an Urban Informal Settlement Area in Nairobi, Kenya. *The American Journal of Tropical Medicine and Hygiene* **2016**, *94*, 122–127. doi:10.4269/ajtmh.15-0293.
303. Mbae, C.; Mulinge, E.; Waruru, A.; Ngugi, B.; Wainaina, J.; Kariuki, S. Genetic Diversity of Cryptosporidium in Children in an Urban Informal Settlement of Nairobi, Kenya. *PLOS ONE* **2015**, *10*, e0142055. doi:10.1371/journal.pone.0142055.
304. Bansal, P.G.; Toteja, G.S.; Bhatia, N.; Vikram, N.K.; Siddhu, A. Impact of weekly iron folic acid supplementation with and without vitamin B12 on anaemic adolescent girls: a randomised clinical trial. *European Journal of Clinical Nutrition* **2015**, *70*, 730–737. doi:10.1038/ejcn.2015.215.
305. Islam, M.A.; Sultana, N.; Ahmed, F.; Rahman, M.M.; Rahman, S.R. ANTIGENIC AND GENETIC CHARACTERIZATION OF INFLUENZA B VIRUSES IN 2012 FROM SLUMS, DHAKA, BANGLADESH. *The Southeast Asian journal of tropical medicine and public health* **2015**, *46*, 611–615.
306. Mitra, M.; Shah, N.; Ghosh, A.; Chatterjee, S.; Kaur, I.; Bhattacharya, N.; Basu, S. Efficacy and safety of vi-tetanus toxoid conjugated typhoid vaccine (PedaTyph^{TM}) in Indian children: School based cluster randomized study. *Human Vaccines & Immunotherapeutics* **2016**, *12*, 939–945. doi:10.1080/21645515.2015.1117715.
307. van der Kop, M.L.; Muhula, S.; Ekström, A.M.; Jongbloed, K.; Smillie, K.; Abunah, B.; Kinagwi, K.; Kyomuhangi, L.B.; Gelmon, L.; Ojakaa, D.I.; Lester, R.T.; Awiti, P.O. Participation in a mobile health intervention trial to improve retention in HIV care: does gender matter? *Journal of Telemedicine and Telecare* **2016**, *23*, 314–320. doi:10.1177/1357633x16643457.

308. Nawagi, F.; Mpimbaza, A.; Mukisa, J.; Serwadda, P.; Kyalema, S.; Kizza, D. Knowledge and practices related to sexually transmitted infections among women of reproductive age living in Katanga slum, Kampala, Uganda. *African Health Sciences* **2016**, *16*, 116. doi:10.4314/ahs.v16i1.15.
309. Malhotra, B.; Dashora, D.; Kumar, V.; Goyal, S.; Sharma, B.; Kumar, M.; Gupta, K.; Sharma, V.; Chauhan, D.; Katoch, K.; Katoch, V. Genetic diversity & drug sensitivity profiles of Mycobacterium tuberculosis isolates from two slums of Jaipur city, Rajasthan, India. *Indian Journal of Medical Research* **2017**, *145*, 74. doi:10.4103/ijmr.ijmr_336_14.
310. Gyang, V.P.; Chuang, T.W.; Liao, C.W.; Lee, Y.L.; Akinwale, O.P.; Orok, A.; Ajibaye, O.; Babasola, A.J.; Cheng, P.C.; Chou, C.M.; others. Intestinal parasitic infections: current status and associated risk factors among school aged children in an archetypal African urban slum in Nigeria. *Journal of Microbiology, Immunology and Infection* **2019**, *52*, 106–113.
311. Gikonyo, J.N.; Nyangao, J.; Mbae, C.; Sang, C.; Njagi, E.; Ngeranwa, J.; Esona, M.; Seheri, M.L.; Gitau, G.W.; Raini, K.; Kariuki, S. Molecular characterization of group A rotaviruses in Mukuru slums Kenya: detection of novel strains circulating in children below 5 years of age. *BMC Research Notes* **2017**, *10*. doi:10.1186/s13104-017-2611-z.
312. Adane, M.; Mengistie, B.; Kloos, H.; Medhin, G.; Mulat, W. Sanitation facilities, hygienic conditions, and prevalence of acute diarrhea among under-five children in slums of Addis Ababa, Ethiopia: Baseline survey of a longitudinal study. *PLOS ONE* **2017**, *12*, e0182783. doi:10.1371/journal.pone.0182783.
313. van der Kop, M.L.; Muhula, S.; Patel, A.; Thabane, L.; Awiti, P.; Kyomuhangi, L.; Abunah, B.; Nagide, P.I.; Smillie, K.; Ojakaa, D.I.; Kimani, J.; Ekström, A.M.; Lester, R.T. Gender differences in health-related quality of life at the time of a positive HIV test - a cross-sectional study in a resource-poor, high prevalence setting in Nairobi, Kenya. *AIDS Care* **2017**, *30*, 493–499. doi:10.1080/09540121.2017.1417970.
314. Harada, H.; Fujimori, Y.; Gomi, R.; Ahsan, M.; Fujii, S.; Sakai, A.; Matsuda, T. Pathotyping of Escherichia coli isolated from community toilet wastewater and stored drinking water in a slum in Bangladesh. *Letters in Applied Microbiology* **2018**, *66*, 542–548. doi:10.1111/lam.12878.
315. Schneider, A.G.; Casanovas-Massana, A.; Hacker, K.P.; Wunder, E.A.; Begon, M.; Reis, M.G.; Childs, J.E.; Costa, F.; Lindow, J.C.; Ko, A.I. Quantification of pathogenic Leptospira in the soils of a Brazilian urban slum. *PLOS Neglected Tropical Diseases* **2018**, *12*, e0006415. doi:10.1371/journal.pntd.0006415.
316. Ziraba, A.; Orindi, B.; Muuo, S.; Floyd, S.; Birdthistle, I.J.; Mumah, J.; Osindo, J.; Njoroge, P.; Kabiru, C.W. Understanding HIV risks among adolescent girls and young women in informal settlements of Nairobi, Kenya: Lessons for DREAMS. *PLOS ONE* **2018**, *13*, e0197479. doi:10.1371/journal.pone.0197479.
317. Winter, S.; Barchi, F.; Dzombo, M.N. Drivers of women's sanitation practices in informal settlements in sub-Saharan Africa: a qualitative study in Mathare Valley, Kenya. *International Journal of Environmental Health Research* **2018**, *28*, 609–625. doi:10.1080/09603123.2018.1497778.
318. Otsuka, Y.; Agestika, L.; Widyarani.; Sintawardani, N.; Yamauchi, T. Risk Factors for Undernutrition and Diarrhea Prevalence in an Urban Slum in Indonesia: Focus on Water, Sanitation, and Hygiene. *The American Journal of Tropical Medicine and Hygiene* **2019**, *100*, 727–732. doi:10.4269/ajtmh.18-0063.
319. Winter, S.; Dzombo, M.N.; Barchi, F. Exploring the complex relationship between women's sanitation practices and household diarrhea in the slums of Nairobi: a cross-sectional study. *BMC Infectious Diseases* **2019**, *19*. doi:10.1186/s12879-019-3875-9.
320. Hulzebosch, A.; van de Vijver, S.; Oti, S.O.; Egondi, T.; Kyobutungi, C. Profile of people with hypertension in Nairobi's slums: a descriptive study. *Globalization and Health* **2015**, *11*. doi:10.1186/s12992-015-0112-1.
321. Werner, M.E.; van de Vijver, S.; Adhiambo, M.; Egondi, T.; Oti, S.O.; Kyobutungi, C. Results of a hypertension and diabetes treatment program in the slums of Nairobi: a retrospective cohort study. *BMC Health Services Research* **2015**, *15*. doi:10.1186/s12913-015-1167-7.
322. Haregu, T.N.; Oti, S.; Ngomi, N.; Khayeka-wandabwa, C.; Egondi, T.; Kyobutungi, C. Interlinkage among cardio-metabolic disease markers in an urban poor setting in Nairobi, Kenya. *Global Health Action* **2016**, *9*, 30626. doi:10.3402/gha.v9.30626.
323. van de Vijver, S.; Oti, S.O.; Gomez, G.B.; Agyemang, C.; Egondi, T.; van Charante, E.M.; Brewster, L.M.; Hankins, C.; Tanovic, Z.; Ezeh, A.; Kyobutungi, C.; Stronks, K. Impact evaluation of a community-based intervention for prevention of cardiovascular diseases in the slums of Nairobi: the SCALE-UP study. *Global Health Action* **2016**, *9*, 30922. doi:10.3402/gha.v9.30922.

324. Banerjee, S.; Bandyopadhyay, L.; Dasgupta, A.; Paul, B.; Chattopadhyay, O. Work Related Musculoskeletal Morbidity among Tailors: A Cross Sectional Study in a Slum of Kolkata. *Kathmandu Univ Med J* **2016**, *56*, 305–10.
325. Groot, H.E.; Muthuri, S.K. Comparison of domains of self-reported physical activity between Kenyan adult urban-slum dwellers and national estimates. *Global Health Action* **2017**, *10*, 1342350. doi:10.1080/16549716.2017.1342350.
326. Asiki, G.; Mohamed, S.F.; Wambui, D.; Wainana, C.; Muthuri, S.; Ramsay, M.; Kyobutungi, C. Sociodemographic and behavioural factors associated with body mass index among men and women in Nairobi slums: AWI-Gen Project. *Global Health Action* **2018**, *11*, 1470738. doi:10.1080/16549716.2018.1470738.
327. Doval, H.; Mariani, J.; Gómez, G.; Vulcano, L.; Parlanti, L.; Gavranovic, M.; Iemma, M.; Sanchez, R.; Macchia, A. Cardiovascular and other risk factors among people who live in slums in Buenos Aires, Argentina. *Public Health* **2019**, *170*, 38–44. doi:10.1016/j.puhe.2019.02.014.
328. Morberg, D.; López, Y.A.; Moreira, S.; Prata, N.; Riley, L.; Peña, M.B. The rheumatic heart disease healthcare paradox: disease persistence in slums despite universal healthcare coverage—a provider perspective qualitative study. *Public Health* **2019**, *171*, 15–23. doi:10.1016/j.puhe.2019.03.015.
329. Donta, B.; Nair, S.; Prakasam, C.; Begum, S. Socio-demographic factors associated with domestic violence in urban slums, Mumbai, Maharashtra, India. *Indian Journal of Medical Research* **2015**, *141*, 783. doi:10.4103/0971-5916.160701.
330. Nasrullah, M.; Zakar, R.; Zakar, M.Z.; Abbas, S.; Safdar, R. Circumstances leading to intimate partner violence against women married as children: a qualitative study in Urban Slums of Lahore, Pakistan. *BMC International Health and Human Rights* **2015**, *15*. doi:10.1186/s12914-015-0060-0.
331. Chakraborty, P.; Daruwalla, N.; Jayaraman, A.; Pantvaidya, S. "You Are a Part of the Solution": Negotiating Gender-Based Violence and Engendering Change in Urban Informal Settlements in Mumbai, India. *Violence Against Women* **2016**, *23*, 1336–1360. doi:10.1177/1077801216659941.
332. Kimemia, D.; van Niekerk, A.; Govender, R.; Seedat, M. Burns and fires in South Africa's informal settlements: Have approved kerosene stoves improved safety? *Burns* **2018**, *44*, 969–979. doi:10.1016/j.burns.2017.11.006.
333. Bennett, R.; Chepngeno-Langat, G.; Evandrou, M.; Falkingham, J. Resilience in the face of post-election violence in Kenya: The mediating role of social networks on wellbeing among older people in the Korogocho informal settlement, Nairobi. *Social Science & Medicine* **2015**, *128*, 159–167. doi:10.1016/j.socscimed.2015.01.033.
334. Korn, A.; Bolton, S.M.; Spencer, B.; Alarcon, J.A.; Andrews, L.; Voss, J.G. Physical and Mental Health Impacts of Household Gardens in an Urban Slum in Lima, Peru. *International Journal of Environmental Research and Public Health* **2018**, *15*, 1751. doi:10.3390/ijerph15081751.
335. Kumar, M.; Amugune, B.; Madeghe, B.; Wambua, G.N.; Osok, J.; Polkonikova-Wamoto, A.; Bukusi, D.; Were, F.; Huang, K.Y. Mechanisms associated with maternal adverse childhood experiences on offspring's mental health in Nairobi informal settlements: a mediational model testing approach. *BMC Psychiatry* **2018**, *18*. doi:10.1186/s12888-018-1953-y.
336. Mutisya, M.; bakwin Kandala, N.; Ngware, M.W.; Kabiru, C.W. Household food (in)security and nutritional status of urban poor children aged 6 to 23 months in Kenya. *BMC Public Health* **2015**, *15*. doi:10.1186/s12889-015-2403-0.
337. Chanani, S.; Wacksman, J.; Deshmukh, D.; Pantvaidya, S.; Fernandez, A.; Jayaraman, A. M-Health for Improving Screening Accuracy of Acute Malnutrition in a Community-Based Management of Acute Malnutrition Program in Mumbai Informal Settlements. *Food and Nutrition Bulletin* **2016**, *37*, 504–516. doi:10.1177/0379572116657241.
338. Goudet, S.M.; Kimani-Murage, E.W.; Wekesah, F.; Wanjohi, M.; Griffiths, P.L.; Bogin, B.; Madise, N.J. How does poverty affect children's nutritional status in Nairobi slums? A qualitative study of the root causes of undernutrition. *Public Health Nutrition* **2016**, *20*, 608–619. doi:10.1017/s1368980016002445.
339. Kimani-Murage, E.W.; Griffiths, P.L.; Wekesah, F.M.; Wanjohi, M.; Muhia, N.; Muriuki, P.; Egondi, T.; Kyobutungi, C.; Ezeh, A.C.; McGarvey, S.T.; Musoke, R.N.; Norris, S.A.; Madise, N.J. Effectiveness of home-based nutritional counselling and support on exclusive breastfeeding in urban

poor settings in Nairobi: a cluster randomized controlled trial. *Globalization and Health* **2017**, *13*. doi:10.1186/s12992-017-0314-9.
340. Macharia, T.N.; Ochola, S.; Mutua, M.K.; Kimani-Murage, E.W. Association between household food security and infant feeding practices in urban informal settlements in Nairobi, Kenya. *Journal of Developmental Origins of Health and Disease* **2018**, *9*, 20–29. doi:10.1017/s2040174417001064.
341. Islam, M.M.; Sanin, K.I.; Mahfuz, M.; Ahmed, A.M.S.; Mondal, D.; Haque, R.; Ahmed, T. Risk factors of stunting among children living in an urban slum of Bangladesh: findings of a prospective cohort study. *BMC Public Health* **2018**, *18*. doi:10.1186/s12889-018-5101-x.
342. Sidze, E.M.; Elungata'a, P.; Maina, B.W.; Mutua, M.M. Does the Quality of Parent-Child Connectedness Matter for Adolescents' Sexual Behaviors in Nairobi Informal Settlements? *Archives of Sexual Behavior* **2014**, *44*, 631–638. doi:10.1007/s10508-014-0402-3.
343. Kimani-Murage, E.W.; Wekesah, F.; Wanjohi, M.; Kyobutungi, C.; Ezeh, A.C.; Musoke, R.N.; Norris, S.A.; Madise, N.J.; Griffiths, P. Factors affecting actualisation of the WHO breastfeeding recommendations in urban poor settings in Kenya. *Maternal & Child Nutrition* **2014**, *11*, 314–332. doi:10.1111/mcn.12161.
344. Wekesa, E.; Coast, E. Contraceptive need and use among individuals with HIV/AIDS living in the slums of Nairobi, Kenya. *International Journal of Gynecology & Obstetrics* **2015**, *130*, E31–E36. doi:10.1016/j.ijgo.2015.05.001.
345. Okigbo, C.C.; Kabiru, C.W.; Mumah, J.N.; Mojola, S.A.; Beguy, D. Influence of parental factors on adolescents' transition to first sexual intercourse in Nairobi, Kenya: a longitudinal study. *Reproductive Health* **2015**, *12*. doi:10.1186/s12978-015-0069-9.
346. Bakibinga, P.; Kamande, E.; Omuya, M.; Ziraba, A.K.; Kyobutungi, C. The role of a decision-support smartphone application in enhancing community health volunteers' effectiveness to improve maternal and newborn outcomes in Nairobi, Kenya: quasi-experimental research protocol. *BMJ Open* **2017**, *7*, e014896. doi:10.1136/bmjopen-2016-014896.
347. Girod, C.; Ellis, A.; Andes, K.L.; Freeman, M.C.; Caruso, B.A. Physical, Social, and Political Inequities Constraining Girls' Menstrual Management at Schools in Informal Settlements of Nairobi, Kenya. *Journal of Urban Health* **2017**, *94*, 835–846. doi:10.1007/s11524-017-0189-3.
348. Stark, L. Early marriage and cultural constructions of adulthood in two slums in Dar es Salaam. *Culture, Health & Sexuality* **2017**, *20*, 888–901. doi:10.1080/13691058.2017.1390162.
349. Jayaweera, R.T.; Ngui, F.M.; Hall, K.S.; Gerdts, C. Women's experiences with unplanned pregnancy and abortion in Kenya: A qualitative study. *PLOS ONE* **2018**, *13*, e0191412. doi:10.1371/journal.pone.0191412.
350. Silva, V.A.; Caminha, M.F.; Silva, S.L.; Serva, V.M.; Azevedo, P.T.; Filho, M.B. Maternal breastfeeding: indicators and factors associated with exclusive breastfeeding in a subnormal urban cluster assisted by the Family Health Strategy. *Jornal de Pediatria* **2019**, *95*, 298–305. doi:10.1016/j.jped.2018.01.004.
351. Shafi, S.; Mohan, U.; Singh, S. Barriers for low acceptance of no scalpel vasectomy among slum dwellers of Lucknow City. *Indian Journal of Public Health* **2019**, *63*, 10. doi:10.4103/ijph.ijph_44_18.
352. Winter, S.C.; Dreibelbis, R.; Dzombo, M.N.; Barchi, F. A mixed-methods study of women's sanitation utilization in informal settlements in Kenya. *PLOS ONE* **2019**, *14*, e0214114. doi:10.1371/journal.pone.0214114.
353. Machiyama, K.; Mumah, J.N.; Mutua, M.; Cleland, J. Childbearing desires and behaviour: a prospective assessment in Nairobi slums. *BMC Pregnancy and Childbirth* **2019**, *19*. doi:10.1186/s12884-019-2245-3.
354. Ajlouni, M.T. Social determinants of health in selected slum areas in Jordan: challenges and policy directions. *The International Journal of Health Planning and Management* **2014**, *31*, 113–125. doi:10.1002/hpm.2267.
355. Corburn, J.; Karanja, I. Informal settlements and a relational view of health in Nairobi, Kenya: sanitation, gender and dignity. *Health Promotion International* **2014**, *31*, 258–269. doi:10.1093/heapro/dau100.
356. Subbaraman, R.; Nolan, L.; Sawant, K.; Shitole, S.; Shitole, T.; Nanarkar, M.; Patil-Deshmukh, A.; Bloom, D.E. Multidimensional Measurement of Household Water Poverty in a Mumbai Slum: Looking Beyond Water Quality. *PLOS ONE* **2015**, *10*, e0133241. doi:10.1371/journal.pone.0133241.
357. Shibata, T.; Wilson, J.L.; Watson, L.M.; Nikitin, I.V.; Ansariadi.; Ane, R.L.; Maidin, A. Life in a landfill slum, children s health, and the Millennium Development Goals. *Science of The Total Environment* **2015**, *536*, 408–418. doi:10.1016/j.scitotenv.2015.05.137.

358. Adams, E.A.; Vásquez, W.F. Do the urban poor want household taps? Community preferences and willingness to pay for household taps in Accra, Ghana. *Journal of Environmental Management* **2019**, *247*, 570–579. doi:10.1016/j.jenvman.2019.06.113.
359. Wilunda, B.; Ng, N.; Williams, J.S. Health and ageing in Nairobi's informal settlements-evidence from the International Network for the Demographic Evaluation of Populations and Their Health (INDEPTH): a cross sectional study. *BMC Public Health* **2015**, *15*. doi:10.1186/s12889-015-2556-x.
360. Mukama, T.; Ndejjo, R.; Musoke, D.; Musinguzi, G.; Halage, A.A.; Carpenter, D.O.; Ssempebwa, J.C. Practices, Concerns, and Willingness to Participate in Solid Waste Management in Two Urban Slums in Central Uganda. *Journal of Environmental and Public Health* **2016**, *2016*, 1–7. doi:10.1155/2016/6830163.
361. Owusu-Ansah, F.E.; Tagbor, H.; Togbe, M.A. Access to health in city slum dwellers: The case of Sodom and Gomorrah in Accra, Ghana. *African Journal of Primary Health Care & Family Medicine* **2016**, *8*. doi:10.4102/phcfm.v8i1.822.
362. Jawhari, B.; Keenan, L.; Zakus, D.; Ludwick, D.; Isaac, A.; Saleh, A.; Hayward, R. Barriers and facilitators to Electronic Medical Record (EMR) use in an urban slum. *International Journal of Medical Informatics* **2016**, *94*, 246–254. doi:10.1016/j.ijmedinf.2016.07.015.
363. Falconi, T.M.A.; Kulinkina, A.V.; Mohan, V.R.; Francis, M.R.; Kattula, D.; Sarkar, R.; Ward, H.; Kang, G.; Balraj, V.; Naumova, E.N. Quantifying tap-to-household water quality deterioration in urban communities in Vellore, India: The impact of spatial assumptions. *International Journal of Hygiene and Environmental Health* **2017**, *220*, 29–36. doi:10.1016/j.ijheh.2016.09.019.
364. Anastasi, E.; Ekanem, E.; Hill, O.; Oluwakemi, A.A.; Abayomi, O.; Bernasconi, A. Unmasking inequalities: Sub-national maternal and child mortality data from two urban slums in Lagos, Nigeria tells the story. *PLOS ONE* **2017**, *12*, e0177190. doi:10.1371/journal.pone.0177190.
365. Thakur, M.; Boudewijns, E.A.; Babu, G.R.; Winkens, B.; de Witte, L.P.; Gruiskens, J.; Sushama, P.; Ghergu, C.T.; van Schayck, O.C.P. Low-smoke chulha in Indian slums: study protocol for a randomised controlled trial. *BMC Public Health* **2017**, *17*. doi:10.1186/s12889-017-4369-6.
366. Ruiz-Díaz, M.S.; Gómez-Camargo, D.E.; Alario, Á.; Salguedo-Madrid, G.I.; Mora-García, G.J. Analysis of Health Indicators in Two Rural Communities on the Colombian Caribbean Coast: Poor Water Supply and Education Level Are Associated with Water-Related Diseases. *The American Journal of Tropical Medicine and Hygiene* **2017**, *97*, 1378–1392. doi:10.4269/ajtmh.16-0305.
367. Aboderin, I.; Kano, M.; Owii, H.A. Toward "Age-Friendly Slums"? Health Challenges of Older Slum Dwellers in Nairobi and the Applicability of the Age-Friendly City Approach. *International Journal of Environmental Research and Public Health* **2017**, *14*, 1259. doi:10.3390/ijerph14101259.
368. Aboderin, I.; Nanyonjo, A. Musculoskeletal health conditions among older populations in urban slums in sub-Saharan Africa. *Best Practice & Research Clinical Rheumatology* **2017**, *31*, 115–128. doi:10.1016/j.berh.2017.11.001.
369. Sushama, P.; Ghergu, C.; Meershoek, A.; de Witte, L.P.; van Schayck, O.C.P.; Krumeich, A. Dark clouds in co-creation, and their silver linings. *Global Health Action* **2018**, *11*, 1421342. doi:10.1080/16549716.2017.1421342.
370. Das, M.; Angeli, F.; Krumeich, A.J.S.M.; van Schayck, O.C.P. The gendered experience with respect to health-seeking behaviour in an urban slum of Kolkata, India. *International Journal for Equity in Health* **2018**, *17*. doi:10.1186/s12939-018-0738-8.
371. George, C.E.; Norman, G.; Wadugodapitya, A.; Rao, S.V.; Nalige, S.; Radhakrishnan, V.; Behar, S.; de Witte, L. Health issues in a Bangalore slum: findings from a household survey using a mobile screening toolkit in Devarajeevanahalli. *BMC Public Health* **2019**, *19*. doi:10.1186/s12889-019-6756-7.
372. Kamau, N.; Njiru, H. Water, Sanitation and Hygiene Situation in Kenyas Urban Slums. *Journal of Health Care for the Poor and Underserved* **2018**, *29*, 321–336. doi:10.1353/hpu.2018.0022.
373. Musoke, D.; Ndejjo, R.; Halage, A.A.; Kasasa, S.; Ssempebwa, J.C.; Carpenter, D.O. Drinking Water Supply, Sanitation, and Hygiene Promotion Interventions in Two Slum Communities in Central Uganda. *Journal of Environmental and Public Health* **2018**, *2018*, 1–9. doi:10.1155/2018/3710120.
374. Aseyo, R.E.; Mumma, J.; Scott, K.; Nelima, D.; Davis, E.; Baker, K.K.; Cumming, O.; Dreibelbis, R. Realities and experiences of community health volunteers as agents for behaviour change: evidence from an informal urban settlement in Kisumu, Kenya. *Human Resources for Health* **2018**, *16*. doi:10.1186/s12960-018-0318-4.

375. Panchang, S.V. Demand for improved sanitation in an urban informal settlement in India: role of the local built environment. *International Journal of Environmental Health Research* **2018**, *29*, 194–208. doi:10.1080/09603123.2018.1533530.

376. van der Heijden, J.; Gray, N.; Stringer, B.; Rahman, A.; Akhter, S.; Kalon, S.; Dada, M.; Biswas, A. 'Working to stay healthy', health-seeking behaviour in Bangladesh's urban slums: a qualitative study. *BMC Public Health* **2019**, *19*. doi:10.1186/s12889-019-6750-0.

Sample Availability: Samples of the compounds ...... are available from the authors.
